# Overcoming resistance to BRAF^V600E^ inhibition in melanoma by deciphering and targeting personalized protein network alterations

**DOI:** 10.1038/s41698-021-00190-3

**Published:** 2021-06-10

**Authors:** S. Vasudevan, E. Flashner-Abramson, Heba Alkhatib, Sangita Roy Chowdhury, I. A. Adejumobi, D. Vilenski, S. Stefansky, A. M. Rubinstein, N. Kravchenko-Balasha

**Affiliations:** The Institute of Biomedical and Oral Research, Hebrew University of Jerusalem, Jerusalem, Israel

**Keywords:** Biophysics, Systems biology, Targeted therapies, Cancer therapeutic resistance

## Abstract

BRAF^V600E^ melanoma patients, despite initially responding to the clinically prescribed anti-BRAF^V600E^ therapy, often relapse, and their tumors develop drug resistance. While it is widely accepted that these tumors are originally driven by the BRAF^V600E^ mutation, they often eventually diverge and become supported by various signaling networks. Therefore, patient-specific altered signaling signatures should be deciphered and treated individually. In this study, we design individualized melanoma combination treatments based on personalized network alterations. Using an information-theoretic approach, we compute high-resolution patient-specific altered signaling signatures. These altered signaling signatures each consist of several co-expressed subnetworks, which should all be targeted to optimally inhibit the entire altered signaling flux. Based on these data, we design smart, personalized drug combinations, often consisting of FDA-approved drugs. We validate our approach in vitro and in vivo showing that individualized drug combinations that are rationally based on patient-specific altered signaling signatures are more efficient than the clinically used anti-BRAF^V600E^ or BRAF^V600E^/MEK targeted therapy. Furthermore, these drug combinations are highly selective, as a drug combination efficient for one BRAF^V600E^ tumor is significantly less efficient for another, and vice versa. The approach presented herein can be broadly applicable to aid clinicians to rationally design patient-specific anti-melanoma drug combinations.

## Introduction

The rates of melanoma have been rapidly increasing (NIH, www.cancer.org). Melanoma is one of the most common cancers in young adults, and the risk for melanoma increases with age (NIH, www.cancer.org). However, alongside the rapid increase in incidence, there has also been rapid clinical advancement over the past decade, with targeted therapy and immunotherapy that have become available to melanoma patients^1^.

Melanoma is associated with a great burden of somatic genetic alterations^2^, with the primary actionable genomic data being an activating mutation in the BRAF gene, BRAF^V600E^, occurring in ~50% of all melanomas^2,3^. Nearly a dozen new treatments have been approved by the Food and Drug Administration (FDA) for unresectable or metastatic melanoma harboring the BRAF^V600E^ mutation, among them vemurafenib (a BRAF^V600E^ inhibitor), cobimetinib (a MEK^MAPK^ inhibitor), or a combination of dabrafenib and trametinib (a BRAF^V600E^ inhibitor and a MEK^MAPK^ inhibitor, respectively)^1^.

While targeted therapy revolutionized melanoma treatment, the high hopes shortly met a disappointment, as it became evident that most patients treated with BRAF^V600E^ inhibitors eventually relapse and their tumors become resistant to the treatment^4–6^. Various combination treatments were suggested to overcome the acquired resistance to BRAF^V600E^ inhibitors^4,5,7,8^. Nevertheless, BRAF^V600E^ and MEK inhibitors remain the only targeted agents approved by the FDA for melanoma. In this study, we design patient-specific targeted treatments for melanoma based on individualized alterations in signaling protein networks, rather than on genomic/protein biomarkers. Attempting to treat patients based on the identification of single biomarkers or signaling pathways may overlook tumor-specific molecular alterations that have evolved during the course of the disease, and the consequently selected therapeutic regimen may lack long-term efficacy resulting from partial targeting of the tumor imbalance. We have shown that different patients may display similar oncogene expression levels, albeit carrying biologically distinct tumors that harbor different sets of unbalanced molecular processes^9^. Therefore, we suggest exploring the cancer data space utilizing an information-theoretic approach that is based on surprisal analysis^9–11^, to unbiasedly identify the altered signaling network structure that has emerged in every single tumor^9,10^.

Our thermodynamic-like viewpoint grasps that tumors are altered biological entities, which deviate from their steady-state due to patient-specific alterations. Those alterations can manifest in various manners that are dependent on environmental or genomic cues (e.g., carcinogens, altered cell–cell communication, mutations, etc.) and give rise to one or more distinct groups of co-expressed oncoproteins in each tumor, named unbalanced processes^9–11^. A patient-specific set of unbalanced processes constitutes a unique signaling signature and provides critical information regarding the elements in this signature that should be targeted. Each tumor can harbor several distinct unbalanced processes, and therefore all of them should be targeted in order to collapse the altered signaling flux in the tumor^10,11^. We have demonstrated that with comprehensive knowledge about the patient-specific altered signaling signature (PaSSS) in hand, we can predict efficacious personalized combinations of targeted drugs in breast cancer^10^.

Herein, we decipher the accurate network structure of co-expressed functional proteins in melanoma tumors, hypothesizing that the PaSSS identified will guide us on how to improve the clinically used BRAF^V600E^-targeted drug combinations. Our aim was to examine the ability of PaSSS-based drug combinations to reduce the development of drug resistance, which frequently develops following BRAF^V600E^ inhibition in melanoma.

To this end, we studied a dataset consisting of 353 BRAF^V600E^ and BRAF^WT^ skin cutaneous melanoma (SKCM) samples, aiming to gain insights into the altered signaling signatures that have emerged in these tumors. A set of 372 thyroid carcinoma (THCA) samples was added to the dataset, as these tumors frequently harbor BRAF^V600E^ as well, therefore enabling studying the commonalities and differences between tumor types that frequently acquire the BRAF^V600E^ mutation.

We show that 17 distinct unbalanced processes are repetitive among the 725 SKCM and THCA patient-derived cancer tissues. Each tumor is characterized by a specific subset of typically 1–3 unbalanced processes. Interestingly, we demonstrate that the PaSSS does not necessarily correlate with the existence of the BRAF^V600E^, namely different tumors can harbor different signatures while both carrying the mutated BRAF, and vice versa—tumors can harbor the same altered signaling signature regardless of whether they carry BRAF^V600E^ or BRAF^WT^. These data suggest that examination of the BRAF gene alone does not suffice to tailor effective medicine to the patient. SKCM and THCA patients harboring BRAF^V600E^ can respond differently to the same therapeutic regimen or rather benefit from the same treatment even though their BRAF mutation status differs.

We experimentally demonstrate our ability to predict effective personalized therapy by analyzing a cell line dataset and tailoring efficacious personalized combination treatments to BRAF^V600E^-harboring melanoma cell lines. The predicted PaSSS-based drug combinations were shown to have an efficacy superior to drug combinations that were not predicted to target the individualized altered signaling signatures, and combinations used in clinics, both in vitro and in vivo. We show that an in-depth resolution of individualized signaling signatures allows inhibiting the development of drug resistance and melanoma regrowth, by demonstrating that while melanoma models develop drug resistance several weeks following initial administration of the clinically used combination, dabrafenib+trametinib, individualized PaSSS-based drug combinations gain a longer-lasting effect and show high selectivity.

## Results

### An overview of the experimental-computational approach

**Fig. 1:**
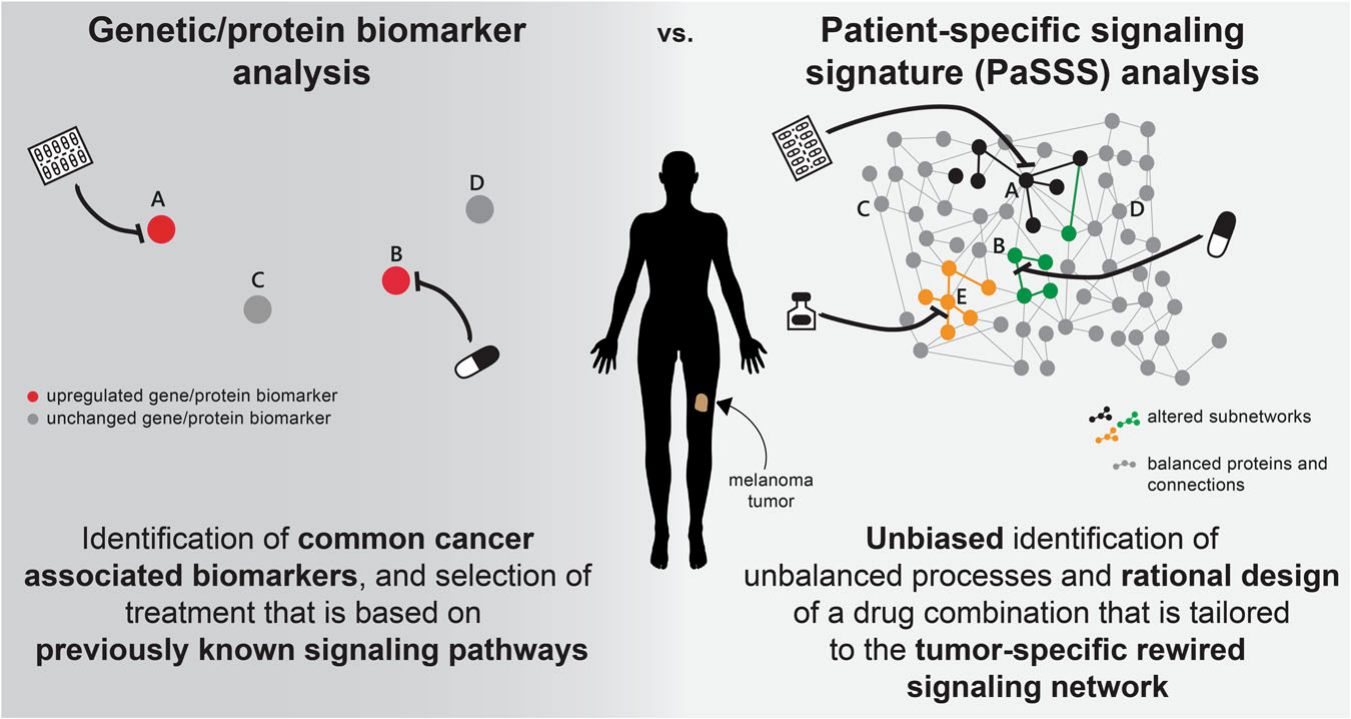
Conventional biomarker analysis vs. patient-specific signaling signature analysis. Genetic/protein biomarker analysis relies on the evaluation of the expression levels of common cancer-type-associated genes or proteins (left). The design of a drug combination is done according to an inference of the state of the surrounding signaling network, based on previous knowledge (left). In contrast, patient-specific signaling signature (PaSSS) analysis involves proteomic analysis of hundreds of cancer-associated proteins, and unbiased identification of the altered signaling signature in every sample, i.e., that does not depend on previous knowledge of melanoma-related signaling pathways. This enables rationally designing personalized combinations of targeted drugs that are based on the patient-specific uniquely rewired signaling network (right).

Biomarker analysis in melanoma relies mainly on the identification of mutations in the BRAF gene^12^. If mutation/upregulation of the mutant BRAF^V600E^ is identified (Fig. 1, left), the patient will likely be treated with a BRAF^V600E^ inhibitor (e.g., vemurafenib^13^ or dabrafenib^14^), possibly concurrently with an inhibitor of MEK^MAPK^ (e.g., trametinib^15^). The combination of BRAF^V600E^ and MEK^MAPK^ inhibitors was shown to be superior to BRAF^V600E^ inhibition alone and to delay or prevent the development of drug resistance^7^. However, the biomarker analysis utilized in clinics lacks information about the altered signaling network, and, for example, may overlook additional or alternative protein targets that, if targeted by drugs, may enhance the efficacy of the treatment (Fig. 1, left).

We utilize an information-theoretic approach that is based on surprisal analysis (see “Methods” section)^9–11^ to gain information regarding the patient-specific signaling signature (PaSSS) that has emerged in every individual tumor (Fig. 1, right). Based on proteomic analysis of the samples, we identify the set of altered protein-protein co-expressed subnetworks, or *unbalanced signaling processes*, that has arisen as a result of constraints (environmental or genomic) that operate on the tumor, and then design a combination of targeted drugs that are predicted to collapse the tumor-specific altered signaling signature (Fig. 1, right and see “Methods” section)^9–11^. We obtained from the TCPA database (The Cancer Proteome Atlas Portal, http://tcpaportal.org) a dataset containing 353 skin cutaneous melanoma (SKCM) and 372 thyroid cancer (THCA) samples (725 samples in total). The thyroid cancer samples were added to the dataset for two main reasons: (1) to increase the number of samples in the dataset, thereby increasing the resolution of the analysis; (2) THCA tumors frequently harbor the BRAF^V600E^ mutation, and we were therefore interested in examining the commonalities and differences between the altered signaling signatures that emerged in SKCM and THCA tumors.

### 17 unbalanced processes repeat themselves throughout 725 SKCM and THCA tumors

The analysis of the dataset revealed that the 725 SKCM and THCA tumors can be described by 17 unbalanced processes (Supp. Fig. 1; the amplitudes for each process in each patient and the importance of each protein in the different processes can be found in Supp. Data 1; the protein composition of each process is presented in Supp. Data 2), i.e., 17 distinct unbalanced processes suffice to reproduce the experimental data (Supp. Fig. 2 and “Methods” section).

**Fig. 2:**
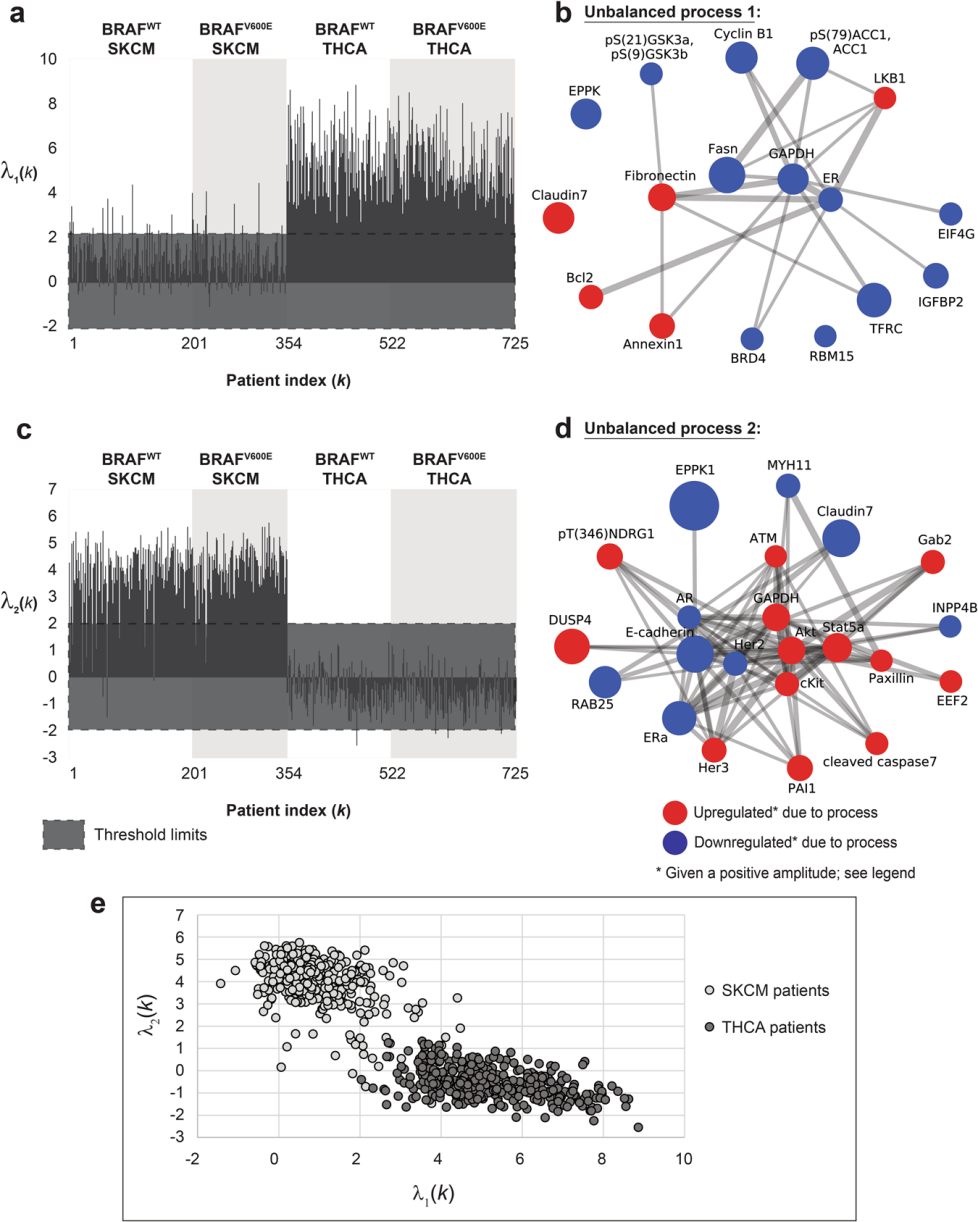
Unbalanced processes 1 and 2 distinguish well between SKCM and THCA tumors when plotted in 2D. The majority of THCA tumors harbor unbalanced process 1 (**a**), while the majority of SKCM tumors harbor unbalanced process 2 (**c**). Unbalanced processes 1 and 2 are shown in panels **b** and **d**. Note that red proteins are upregulated, and blue proteins are downregulated given that the amplitude of the process is positive. In tumors where the amplitude is negative, the direction of change is opposite. **e** A 2D plot showing *λ*_2_(*k*) against *λ*_1_(*k*) for all SKCM and THCA patients. The plot shows nicely the separation between SKCM and THCA patients in this 2D space. Note, however, that every tumor is characterized by a set of unbalanced processes (a PaSSS), and that unbalanced processes 1 and 2 alone do not suffice to describe the complete tumor-specific altered signaling signatures.

Unbalanced processes 1 and 2, the two most significant unbalanced processes, which appear in the largest number of tumors, distinguish well between SKCM and THCA tumors, as can be seen by the 2D plots of *λ*_*α*_(*k*) values (i.e., amplitudes of each process in every tumor; Fig. 2a, c, e). Unbalanced process 1 (Supp. Data 2) appears almost exclusively in THCA tumors (372 THCA tumors harbor unbalanced process 1, vs. 46 SKCM tumors; Fig. 2a,e), while unbalanced process 2 characterizes almost exclusively SKCM tumors (331 SKCM tumors harbor unbalanced process 2 vs. only 4 THCA tumors; Fig. 2c, e). Unbalanced process 1 involves upregulation of proteins that have been previously linked to THCA: LKB1^16^, fibronectin^17,18^, Bcl-2^19^, claudin 7^20^ (Fig. 2b). Unbalanced process 2 is characterized by the upregulation of proteins that have been implicated in melanoma, such as Stat5α^21^, Akt^22^, cKit^23^, Her3^24^, and ATM^25^ (Fig. 2d). As can be seen in the graph in Fig. 2c, unbalanced process 2 was assigned a positive amplitude in all 331 SKCM tumors in which it appears, while in 4 THCA tumors it was assigned a negative amplitude (see also Supp. Data 1). This means that the proteins that participate in this unbalanced process deviate to opposite directions in the two types of tumors (importantly, this remark denotes only the partial deviation that occurred in these proteins due to unbalanced process 2; some of these proteins may have undergone additional deviations due to the activity of other unbalanced processes. See Supp. Data 2 and “Methods” section). Although unbalanced process 2 appears in a significant number of BRAF^V600E^ SKCM patients (Fig. 2c, d), it does not include pS(445)BRAF and downstream signaling. This finding corresponds to a recent characterization of melanoma tissues^3^ and suggests that the signaling signatures of BRAF^V600E^ tissues may diverge over time and acquire additional signaling routs which are not necessarily related to the original driver mutations, such as BRAF^V600E^ or its downstream MEK^MAPK^ signaling.

**Fig. 3:**
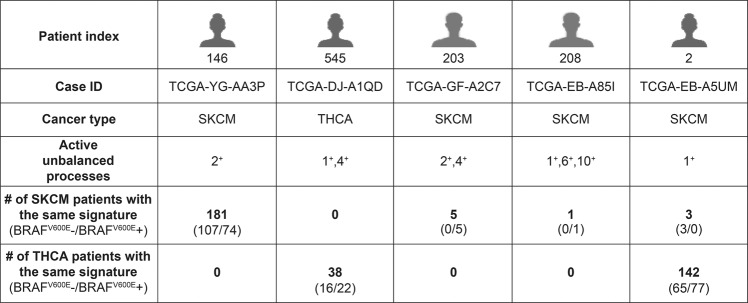
Examples for patient-specific sets of active unbalanced processes. Each patient typically harbors a set of 1–3 active unbalanced processes. Our results show that a specific set of active processes does not necessarily distinguish between BRAFV600E− and BRAFV600E+ patients, or between SKCM and THCA patients.

Unbalanced process 2 can also be found in BRAF^WT^ patients (Fig. 2c). See, for example, patient TCGA-YG-AA3P (Fig. 3). The signature of this patient did not include additional processes. A total of 181 SKCM patients harbor this signaling signature, consisting only of unbalanced process 2: 107 of them harbor BRAF^WT^, and 74 of them harbor BRAF^V600E^ (Fig. 3). In contrast, no THCA patients harbor this signature (Fig. 3). The finding that BRAF^WT^ and BRAF^V600E^ SKCM patients can, in some cases, harbor the same altered signature suggests that these patients can also benefit from the same combination of targeted drugs.

Although unbalanced processes 1 and 2 distinguish well between SKCM and THCA patients (Fig. 2a, c, e), these processes alone do not suffice to describe the PaSSS of all patients. Our analysis suggests that to decipher the altered signaling signature in every patient, 17 unbalanced processes should be considered. Hence, 2D plots may overlook important therapeutic information. When we inspect the patients in the context of a 17-dimensional space, where each dimension represents an unbalanced process, we find that not all SKCM patients harbor unbalanced process 2 and that those who do harbor this process may harbor additional unbalanced processes as well (Fig. 3, Supp. Fig. 3 and Supp. Data 1). We have shown that mapping the patients into a multi-dimensional space (a 17D space in our case) allows deciphering *the set* of unbalanced processes, namely the PaSSS, in *every* tumor. This mapping is crucial for the design of efficacious treatments^10^.

The SKCM patient TCGA-GF-A2C7, for example, is characterized by a PaSSS consisting of unbalanced processes 2 and 4 (Fig. 3). Only 5 SKCM patients were found to be characterized by this set of unbalanced processes, all of which harbor BRAF^V600E^ (Fig. 3).

The SKCM patient TCGA-EB-A85I was found to harbor a PaSSS consisting of unbalanced processes 1, 6, and 10 (Fig. 3). This patient harbors a one-of-a-kind tumor, as no other patients in the dataset harbor this altered signaling signature (Fig. 3).

The PaSSS of THCA patient TCGA-DJ-A1QD includes unbalanced processes 1 and 4 (Fig. 3). This signature characterizes 38 THCA patients, 16 of them BRAF^WT^ and 22 of them BRAF^V600E^ (Fig. 3). These THCA patients may benefit from a combination of drugs that target central protein nodes in unbalanced processes 1 and 4, regardless of whether they harbor BRAF^V600E^ or not. No SKCM patients harbor this altered signaling signature (Fig. 3).

Another interesting finding is that SKCM and THCA patients may harbor the same PaSSS, as is the case of the signature consisting of unbalanced process 1, shared by 3 SKCM patients and 142 THCA patients (Fig. 3 and Supp. Data 1). All these patients may be treated with the same drug combination, targeting key proteins in unbalanced process 1, e.g., LKB1 and fibronectin (Fig. 2b).

### The altered signaling signatures identified in SKCM and THCA are almost mutually exclusive

**Fig. 4:**
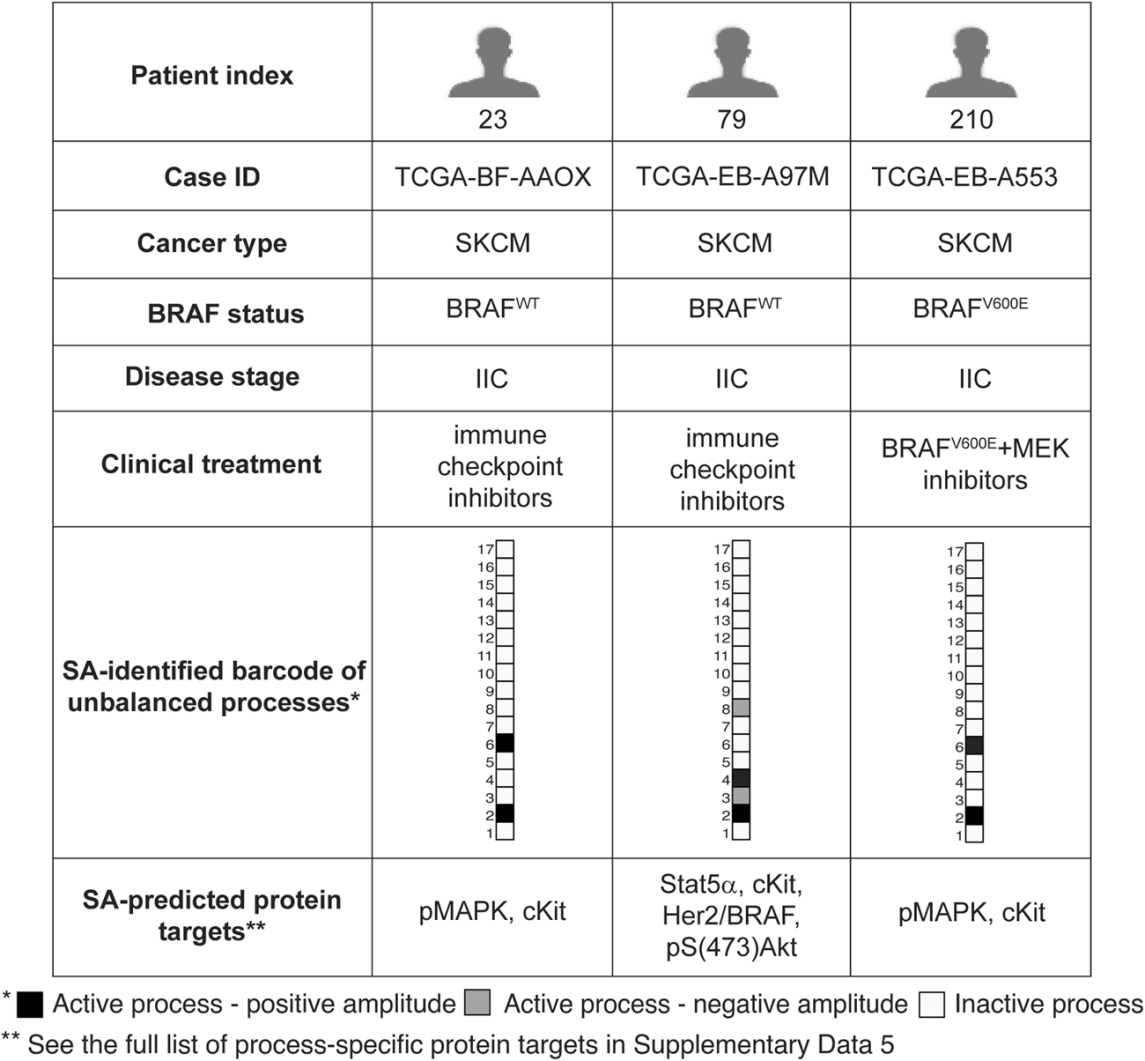
Patient-specific altered signaling signatures, or barcodes, can guide the design of personalized combination therapies. For each tumor, processes with amplitudes exceeding the threshold values (see “Methods” section) were selected and included in patient-specific sets of unbalanced processes. Those sets were converted into schematic barcodes. The sign of the amplitude denotes the direction of the imbalance, i.e., the same unbalanced process can deviate to opposite directions in different patients. Central upregulated proteins from each process were suggested as potential targets for personalized drug combinations.

To explore the entire dataset in terms of the set of unbalanced processes that each patient harbors, we assigned to each patient a patient-specific barcode, denoting the PaSSS, i.e., the set of active unbalanced processes in the specific tumor (Fig. 4, Supp. Data 3). These barcodes represent the mapping of every patient to a 17-dimensional space where each dimension denotes a specific unbalanced process^9,10^. We found that 138 distinct barcodes repeated themselves in the dataset (Supp. Data 4). Interestingly, the barcodes are almost mutually exclusive: 87 of the barcodes characterize SKCM tumors; 84 of them characterize only SKCM tumors and are not harbored by any THCA tumor (Supp. Data 4). 54 barcodes characterize THCA tumors; of them, 51 characterize solely THCA tumors (Supp. Data 4). Most of the barcodes are rare: 81 barcodes are shared by only 5 SKCM tumors or less; 56 of them describe single, one-of-a-kind SKCM tumors (Supp. Data 4). 47 barcodes are shared by only 5 THCA tumors or less; 36 of them describe single THCA tumors (Supp. Data 4). This finding corroborates with our previous studies of signaling signatures in cancer^10^ and underscores the need for personalized cancer diagnosis that is not biased by, e.g., the anatomical origin of the tumor.

### Patient-specific barcodes guide the rational design of personalized targeted combination therapy

We have previously shown the predictive power of our analysis in determining effective patient-tailored combinations of drugs that target key proteins in every unbalanced process^10,11^. Utilizing the maps of the unbalanced processes identified in the dataset herein (Supp. Fig. 1), we predicted process-specific protein targets for each process (Supp. Data 5). Each individual patient is predicted to benefit from a therapy that combines drugs against all the unbalanced processes active in the specific tumor (Fig. 4, Supp. Data 5).

As mentioned above, SKCM patients can in some cases benefit from the same combination therapy, regardless of their BRAF mutational status. This is the case for patients TCGA-EB-A553 (carrying BRAF^V600E^) and TCGA-BF-AAOX (carrying BRAF^WT^), that were found to harbor tumors characterized by the same barcode of unbalanced processes and were therefore predicted to benefit from the same treatment, where pMAPK and cKit are targeted simultaneously (Fig. 4). Patient TCGA-EB-A97M carries BRAF^WT^, as does patient TCGA-BF-AAOX (Fig. 4). However, unbalanced process 6, which is active in patient TCGA-BF-AAOX, is inactive in patient TCGA-EB-A97M (Fig. 4). In addition, patient TCGA-EB-A97M harbors three active unbalanced processes that are not active in the tumor of patient TCGA-BF-AAOX–processes 3, 4, and 8 (Fig. 4). Therefore, the list of proteins that should be targeted in order to collapse the tumor differs in these patients (Fig. 4).

We obtained from the GDC Data Portal (https://portal.gdc.cancer.gov/) data regarding genomic mutations that often occur in SKCM^26^ (Supp. Data 6). We selected 6 mutually exclusive mutations (including BRAF^V600E^, Supp. Fig. 4). We found that SKCM patients harboring the same genomic mutations were characterized by various barcodes according to PaSSS analysis (Supp. Fig. 4) and may thus demand distinct treatments. This result supports the notion that analysis of genomic biomarkers alone may overlook patient-specific aberrations.

### A375 and G361 BRAF-mutated melanoma cell lines harbor distinct altered signaling signatures

To experimentally validate our hypothesis that BRAF^V600E^ harboring cells may benefit from drug combinations that are designed based on the PaSSS identified at the time of diagnosis, we turned to analyze a different dataset containing 290 cell lines originating from 16 types of cancer, including blood, bone, breast, colon, skin, uterus, and more (see “Methods” section). The cell lines were each profiled for the expression levels of 216 proteins and phosphoproteins using a reverse-phase protein assay (RPPA).

PaSSS analysis of this cell line dataset revealed that 17 unbalanced processes were repetitive in the 291 cell lines (Supp. Data 7, Supp. Data 8, Supp. Fig. 5 and “Methods” section).

We randomly selected two melanoma cell lines, A375 and G361, for experimental validation. Both cell lines harbor the mutated BRAF^V600E^. In the clinic, patients bearing tumors with BRAF^V600E^ would all be treated similarly, with BRAF inhibitors alone or in combination with MEK inhibitors^7,15^.

**Fig. 5:**
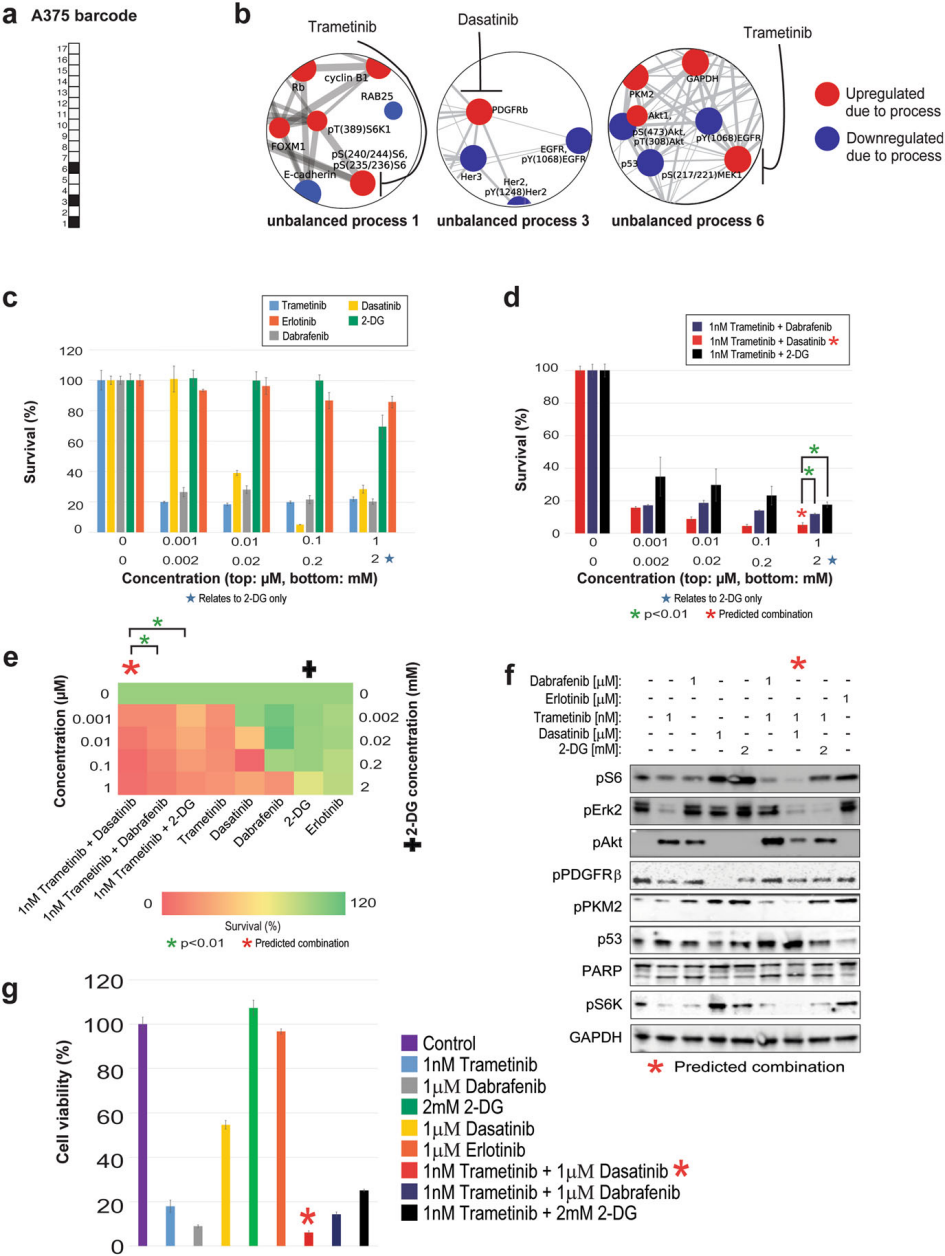
A375 melanoma cells altered signaling signature and SA-based treatment. Even though A375 cells harbor BRAF^V600E^, as do G361 cells, they were found to be characterized by a different set of active unbalanced processes, or PaSSS. **a** Barcode representing the PaSSS of A375 cells, namely the set of active unbalanced processes based on PaSSS analysis. **b** Zoom-in images of the unbalanced processes active in A375 cells, and the drugs targeting the central proteins in each process. The upregulated proteins are colored red and the downregulated proteins are colored blue. **c**, **d** Survival rates of cells in response to different therapies. The cells were treated with the predicted combination (*) to target A375, the treatments used in the clinics for BRAF mutated melanoma malignancies, monotherapies of each treatment and the predicted combination used to target BRAF mutated melanoma cell line G361. The combination predicted to target A375 was more efficient than any other treatment. **e** Results of the survival assay (shown in panels **c** and **d**) are shown as a heatmap. **f** Western blot results after treatment with different therapies. The predicted combination depletes the signaling in A375 cells as represented by a decrease in phosphorylation levels of pS6, pERK, pAkt, pPKM2, and pPDGFRβ. Akt remains active when the cells are treated with monotherapies-trametinib or dabrafenib, and the combination therapies-dabrafenib + trametinib or trametinib + 2-deoxyglucose, the predicted combination of G361. **g** A375 cells were treated as indicated for 72 h and then the viability of the cells was measured in an MTT assay. The effect of the predicted combinations (marked in the figure with asterisk signs) was superior to combinations and single drugs expected to partially inhibit the cell line-specific altered signaling signature.

**Fig. 6:**
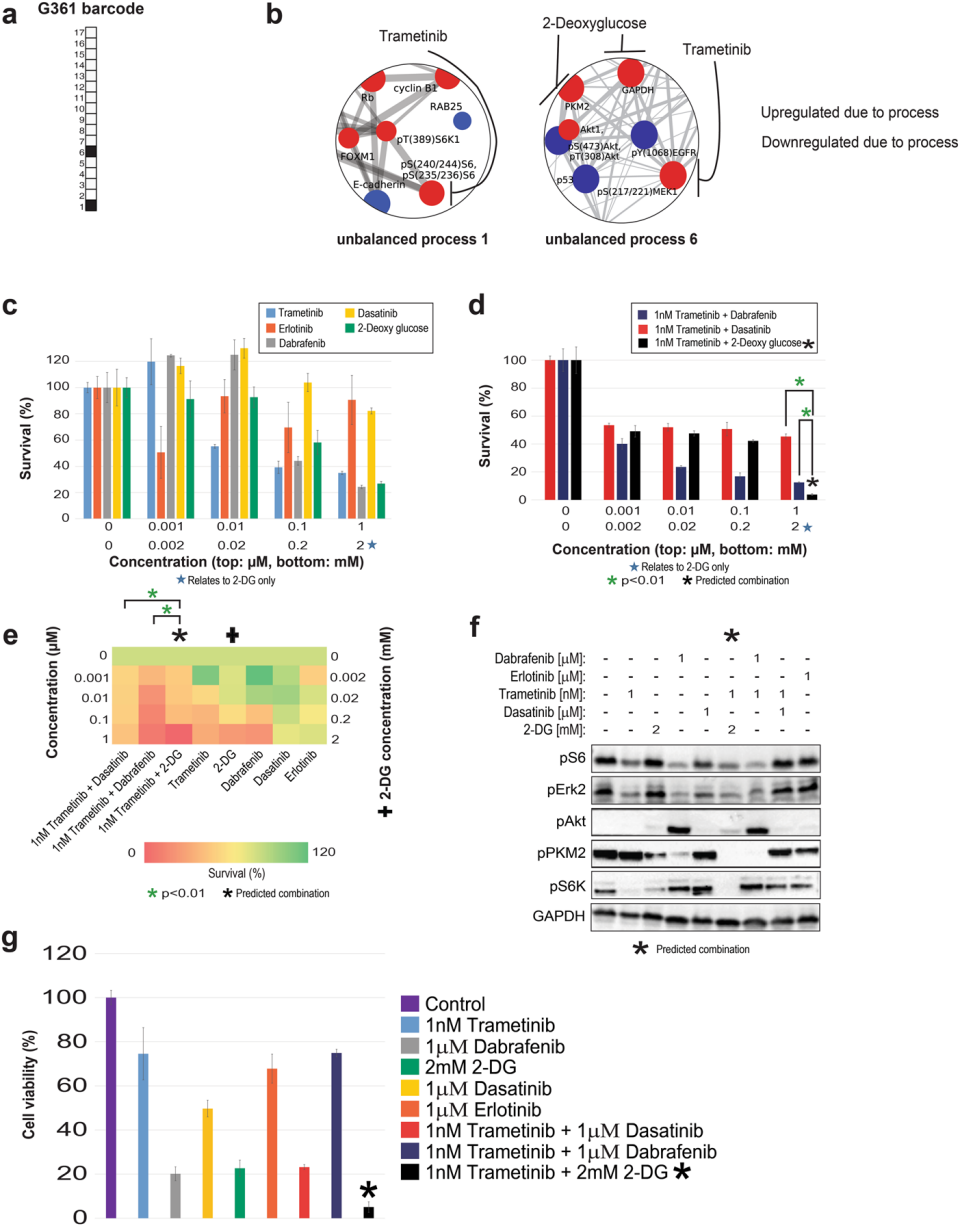
G361 melanoma cells altered signaling signature and treatment. **a** Barcode representing the PaSSS of G361 cells, namely the set of active unbalanced processes identified by PaSSS analysis. **b** Zoom-in images of the unbalanced processes active in G361 cells, and the drugs targeting the central proteins in each process. The upregulated proteins are colored red and the downregulated proteins are colored blue. **c**, **d** Survival rates of cells in response to different therapies. The cells were treated with the predicted combination (*) to target G361, the treatments used in the clinics for BRAF mutated melanoma malignancies, monotherapies of each treatment and the predicted combination used to target BRAF mutated melanoma cell line A375. The combination predicted to target G361 was more efficient than any other treatment. **e** Results of the survival assay (shown in panels **c** and **d**) are shown as a heatmap. **f** Western blot results after treatment with different therapies. The predicted combination depletes the signaling in G361 cells as represented by a decrease in phosphorylation levels of pS6, pERK, and pAkt. Akt remains active when the cells are treated with dabrafenib or dabrafenib + trametinib. **g** G361 cells were treated as indicated for 72 h and then the viability of the cells was measured in an MTT assay. The effect of the predicted combination (marked in with an asterisk sign) was superior to combinations and single drugs expected to partially inhibit the cell line-specific altered signaling signature.

Our analysis, however, shows that A375 and G361 each harbor a distinct PaSSS (Figs. 5 and 6). The PaSSS of A375 consisted of three unbalanced processes, 1, 3, and 6 (Fig. 5a). G361, on the other hand, was found to harbor a PaSSS consisting of unbalanced processes 1 and 6 (Fig. 6a).

Since all of the proteins that participate in a certain unbalanced process undergo coordinated changes and the vast majority of them are functionally connected based on STRING (STRING database) (Supp. Fig. 5), we assumed that targeting one or two central nodes in a process should suffice to inhibit the altered signaling flux through the specific unbalanced process. We further hypothesized that an effective drug combination should consist of drugs that, together, target all the unbalanced processes that are active in the tumor. We have recently demonstrated that targeting one central node leads to reduced flux through the process in which it participates, while leaving other processes essentially unaffected^11^. Therefore, we searched unbalanced processes 1, 3, and 6 for upregulated central nodes that can be targeted by drugs, preferably FDA-approved (The full lists of proteins that participate in the different unbalanced processes are presented in Supp. Data 8, and the images of the unbalanced processes, including the functional connections according to STRING can be found in Supp. Fig. 5). An upregulation of pMEK1/2, GAPDH, and PKM2 was attributed to unbalanced process 6 (Fig. 5b, Fig. 6b), while unbalanced process 3 was characterized by an upregulation of PDGFRβ (Fig. 5b), and unbalanced process 1 involved upregulation of pS6K and pS6 (Fig. 5b, Fig. 6b). We, therefore, predicted for A375 cells that a combination of trametinib (a pMEK1/2 inhibitor, commonly used for melanoma in clinics; also inhibits pS6^27,28^) and dasatinib (a multi-kinase inhibitor targeting also PDGFRβ) should effectively target the three unbalanced processes that constitute the PaSSS of these cells (Fig. 5b). The selection of a multi-kinase inhibitor, dasatinib, to inhibit PDGFRβ instead of a more specific kinase inhibitor was motivated by reports showing that induced expression of certain biomarkers, such as PRKCA and CAV1, was associated with the efficient activity of dasatanib in tissues^29,30^. PKC and CAV1 were associated with unbalanced process 3 along with PDGFRβ, and therefore we selected dasatinib to target this unbalanced process.

Based on the PaSSS of G361, trametinib should effectively target both unbalanced processes, 1 and 6 (Fig. 6b). However, unbalanced process 6 was assigned a relatively high amplitude in G361 cells (Supp. Data 7). Thus, we decided to combine trametinib with another inhibitor that will target additional central nodes in unbalanced process 6: 2-deoxy-D-glucose (2-DG; a glycolysis inhibitor, therefore affecting GAPDH and PKM2; Fig. 6b).

### The predicted drug combinations are cell line-specific and highly efficacious

In A375 cells, trametinib, dabrafenib, and dasatinib killed up to ~80% of the cells, when administered as monotherapies in a range of concentrations between 1 nM and 1 µM (Fig. 5c, e). Erlotinib was used as a negative control, as it was predicted not to target any major node in the PaSSS of A375 cells, and indeed killed only up to ~15% of the cells (Fig. 5c, e). 2-DG, which was predicted to only partially target the unbalanced flux, namely one of the three unbalanced processes active in A375 cells (unbalanced process 6; Fig. 5a, b), killed up to ~30% of the cells when administered as monotherapy (Fig. 5c, e). The clinically used drug combination, trametinib and dabrafenib, was more effective than each drug alone and killed up to ~85% of the cells (Fig. 5d, e). However, we predicted that the clinically used combination would not be optimal in A375 cells, because neither trametinib nor dabrafenib was predicted to target unbalanced process 3 (Fig. 5a, b). Indeed, when trametinib and dabrafenib were administered to the cells, pPDGFRβ was not inhibited (Fig. 5f), suggesting that unbalanced process 3 remained active in A375 cells (Fig. 5b). Interestingly, the combination of trametinib and dabrafenib, or trametinib alone, also invoked an upregulation of pAkt (Fig. 5f). We hypothesize that this can be explained by the fact that pAkt is anti-correlated to pMEK in unbalanced process 6 (Fig. 5b), and therefore in cases where the unbalanced flux is only partially inhibited, the levels of pAkt can increase when pMEK is inhibited. This result corresponds to the previous findings showing that MEK inhibitors may induce Akt activation^31^. Dasatinib, however, abolished the functional activity of PDGFRβ, but did not decrease the levels of pS6 and pS6K from process 1, pERK2 (MEK substrate that participates in process 6; Supp. Data 8) and pPKM2 from process 6 (Fig. 5f), suggesting that only unbalanced process 3 was inhibited by dasatinib (Fig. 5b). p53 is anti-correlated to pMEK in unbalanced process 6 (Fig. 5b) and was upregulated as well when trametinib was added to A375 cells (Fig. 5f).

Overall, these results strengthen the notion of the independence of the unbalanced processes in A375 cells and underscore the need for concurrent inhibition of patient-specific active unbalanced processes in cancer. Indeed, our predicted combination for A375, trametinib, and dasatinib (Fig. 5b), was highly efficacious and killed up to ~95% of the cells (Fig. 5d, e). Trametinib and dasatinib, when combined, diminished pS6, pERK, pS6K, and pPKM2 signaling, lowered the levels of pPDGFRβ, and increased p53 levels (Fig. 5f).

We tested the effect of the combination predicted for G361 cells, trametinib and 2-DG, on A375 cells, and found that it was less effective in inhibiting the intracellular signaling (Fig. 5f), and in inhibiting cell survival (Fig. 5d, e) as compared with the drug combination predicted specifically for the PaSSS of A375. We assume that leaving certain elements in the unbalanced signaling untargeted may not only enrich the cells/subpopulations harboring the untargeted processes but also invoke other, previously undetected pathways (e.g., subpopulations that were initially small and undetectable, and increased during treatment, or rather formed anew during treatment), thereby leading to a switch from one signaling state to another.

In G361 cells, trametinib and 2-DG, both predicted by PaSSS analysis to target the unbalanced signaling flux in G361 cells, demonstrated efficient killing of G361 cells, achieving up to ~65% and ~75% killing, respectively, when administered to the cells as monotherapies at 1 µM (trametinib) and 1 mM (2-DG) (Fig. 6c, e). Dasatinib, which was highly effective in A375 cells, demonstrated a very weak effect in G361 cells, killing only ~20% of the cells when administered at 1 µM (Fig. 6c,e). Erlotinib was used as a negative control, as it was not expected to target any of the unbalanced processes active in G361 cells (Fig. 6b), and indeed killed only up to ~10% of the cells (Fig. 6c, e).

When we tested combinations of drugs, we found that when G361 cells were treated with a combination of trametinib and dabrafenib, the combination was superior to each drug administered alone, and reached ~90% killing of the cells when both drugs were administered at 1 µM (Fig. 6d, e). However, despite the relatively strong effect, this combination evoked pAkt (Fig. 6f), suggesting that some altered signaling pathways remained active in the cells. The results of our analysis denoted that unbalanced process 6 was active with a relatively high amplitude in G361 cells (Supp. Data 7). We, therefore, assumed that the reason that the combination of trametinib and dabrafenib did not abolish the unbalanced signaling flux entirely is that unbalanced process 6 was not effectively shut off, allowing some metabolic activity, and possibly signaling rearrangements. We predicted that the addition of 2-DG to trametinib will more effectively collapse the PaSSS that emerged in G361 cells, because of 2-DG targets GAPDH and PKM2, two additional central upregulated nodes in unbalanced process 6 (Fig. 6b). We indeed found that the combination of trametinib and 2-DG abolished the cells almost completely when trametinib and 2-DG were added at 1 µM and 2 mM, respectively (Fig. 6d, e). The combination of trametinib and 2-DG also effectively turned off the cellular signaling, as represented by the inhibition of pS6, pAkt, pS6K, and pERK, while each drug alone or other combinations we tested failed to do so, leaving some of the elements of the signaling active (Fig. 6f). For example, 2-DG alone reduced pPKM2 levels but did not influence the levels of pS6 and pERK (Fig. 6f), suggesting that process 1 remained active and process 6 was only partially inhibited (Fig. 6b).

When tested in an MTT assay (assessing metabolic activity of the cells), the predicted combinations demonstrated higher efficacy and selectivity and were superior to other drug combinations or to each inhibitor alone (Figs. 5g, 6g). Interestingly, note that while the survival assay shows that treatment of G361 cells with dabrafenib+trametinib resulted in ~90% killing of the cells (Fig. 6d, e), the results of the MTT assay showed that the treated cells remained highly metabolically active (Fig. 6f), suggesting that the treatment with dabrafenib + trametinib leaves the living cells viable. The PaSSS-based prediction, trametinib + 2-DG, however, led to significant inhibition of G361 cell survival, as well as viability (Fig. 6d,e,g).

### As opposed to common therapies used in clinics, the rationally designed cell line-specific drug combinations prevented the development of drug resistance in vitro

**Fig. 7:**
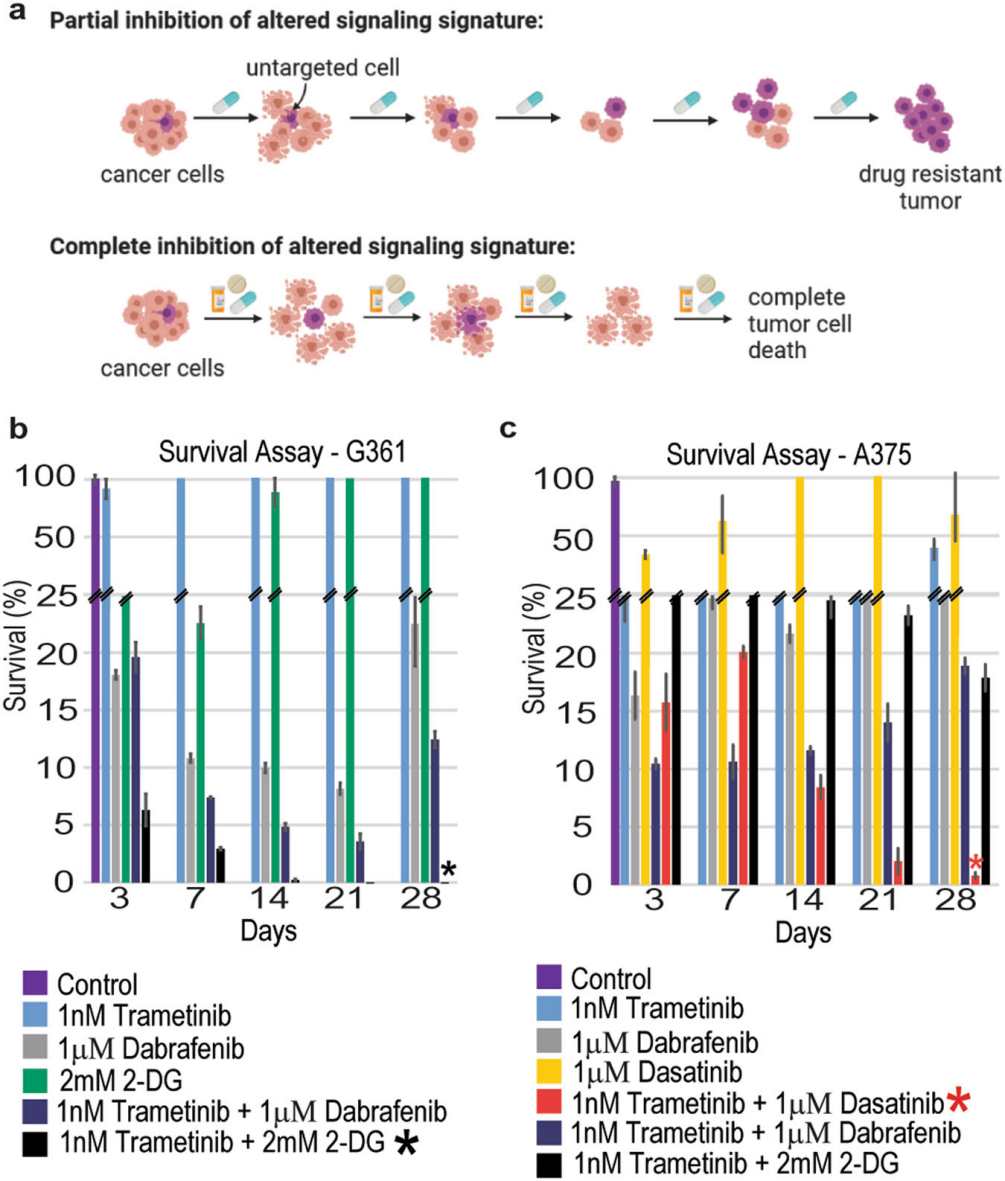
Development of resistance to different therapies. **a** The development of resistance to different types of therapies is shown in the illustration. The cells were treated with different therapies twice a week and then checked for cell survival. **b** G361 cells were treated with monotherapies, dabrafenib + trametinib, or trametinib + 2-DG, twice weekly for 28 days. The cells exhibited signs of drug resistance after 28 days. However, resistance development was not evident in cells that were treated with trametinib + 2-DG. **c** A375 cells were treated with the monotherapies, trametinib + dasatinib, dabrafenib + trametinib, or trametinib + 2-DG, twice weekly for 28 days. Development of resistance was evident after 21 days, but not in cells treated with trametinib + dasatinib.

We hypothesized that since our predicted drug combinations target the main altered processes simultaneously, they may delay or prevent the development of drug resistance (Fig. 7a). To test this hypothesis, G361 and A375 cells were treated twice a week with single inhibitors or with different combinations of inhibitors, for 4 weeks.

In G361 cells, 1 nM of trametinib demonstrated little to no effect on the survival of the cells (Fig. 7b). 1 µM of dabrafenib killed up to ~92% of the cells at day 21, and then the cells began to regrow, even though the drug was still administered to the cells twice a week (Fig. 7b). 2 mM of 2-DG killed up to ~78% of the cells at day 7, and then the cells began to regrow regardless of the presence of the drug (Fig. 7b). Combined treatment with trametinib and dabrafenib, a combination expected to partially target the altered signaling signature (Fig. 6a, b), effectively killed up to ~96% of the cells at day 21, but then the cells began to regrow at day 28 in the presence of the drugs (Fig. 7b). However, when the cells were treated with the G361 PaSSS-based combination, trametinib, and 2-DG (Fig. 6a, b), the cells continued to die until they reached a plateau at day 14, and no regrowth of the cells was evident (Fig. 7b).

Similar results were obtained in A375 cells - all monotherapies led to cellular regrowth after several weeks of treatment (Fig. 7c). Combined treatment with trametinib and dabrafenib achieved 88% killing at day 3, but then the cells grew until they reached ~20% survival at day 28 (Fig. 7c). Trametinib and 2-DG killed 55% of the cells at day 3 with an increase in effect over time, reaching ~18% survival at day 28 (Fig. 7c). The A375 PaSSS-based combination, trametinib, and dasatinib (Fig. 5a, b), demonstrated a significant killing effect that became stronger with time, reaching near complete killing of the cells at 28 days (Fig. 7c).

These results clearly show that the PaSSS-based combinations predicted for each melanoma cell line prevent cellular regrowth in-vitro. Thus, targeting the actual altered signaling state, identified in the melanoma cells, and not necessarily the primary driver mutations, can be especially effective in disturbing the signaling flux and preventing cellular regrowth.

### The predicted drug combinations were superior to clinically used therapies in vivo

**Fig. 8:**
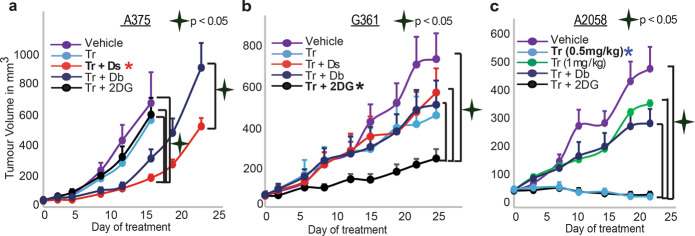
SA-based drug combinations demonstrated significantly reduced tumor growth in vivo. A375 (**a**), G361 (**b**), and A2058 (**c**) cells were injected subcutaneously into mice, and once tumors reached 50 mm^3^, treatments were initiated. In all cases, the PaSSS-based drug combinations predicted to target the cell line-specific altered signaling signature, significantly inhibited tumor growth and demonstrated an effect superior to drug monotherapies/combinations predicted to partially target the PaSSS. Asterisk (*) denotes the cell-specific drug combination predicted for each cell line (red for A375, black for G361, and blue for A2058).

We turned to examine the effect of the PaSSS-predicted drug combination in murine models. The cells were injected subcutaneously into NSG mice, and then treated 6 times a week for up to 4 weeks (Fig. 8; Supp. Fig. 7 shows that the mice demonstrated no significant weight loss during treatment).

A375 tumors that were treated with trametinib alone or with the combination trametinib + 2-DG (predicted to be efficient for G361 but not for A375 cells (Figs. 5, 6)) demonstrated slightly reduced growth relative to vehicle-treated tumors (Fig. 8a). When A375 tumors were treated with the clinically used combination, trametinib + dabrafenib, a stronger effect was observed (Fig. 8a). PaSSS analysis predicted that trametinib + dabrafenib would achieve partial inhibition of the altered signaling in A375 cells (Fig. 5a, b) and that adding dasatinib to trametinib should achieve a more efficient inhibition of intracellular signaling that have emerged in A375 cells (Fig. 5a, b). Indeed, the combination trametinib + dasatinib demonstrated an effect superior to all other treatments and significantly inhibited the growth of A375 tumors (Fig. 8a, Supp. Fig. 6e).

Trametinib alone, or in combination with dasatinib or dabrafenib, was predicted to partially target the PaSSS of G361 cells (Fig. 6a, b). And indeed, these treatments demonstrated a reduction in tumor growth relative to vehicle treatment (Fig. 8b). However, the PaSSS-based combination, trametinib + 2-DG, demonstrated the strongest effect, achieved significant inhibition of G361 tumor growth (Fig. 8b), and reduced the signaling flux (Supp. Fig. 6c).

To further validate the PaSSS-based concept presented in this study we selected an additional BRAF^V600E^ cell line, A2058. The signaling signature of A2058 consists of a single unbalanced process, unbalanced process 1 (Supp. Data 7, 9), which is active in A375 and G361 as well (unbalanced process 1 is represented by the level of the central node, pS6K, in Supp. Fig. 6a). In contrast, process 6 (active in G361 and A375; represented by pMEK) and process 3 (active in A375; represented by PDGFR) were not found to be active in A2058 (Supp. Fig. 6a, Supp. Data 7 and 9). Thus, we predicted that A2058 malignancy should be treated with trametinib monotherapy. Figure 8c demonstrates that a low concentration of trametinib (0.5 mg/kg) was most effective (also Supp. Fig. 6d), and intriguingly more effective than a higher concentration of trametinib (1 mg/kg, Fig. 8c), corresponding to previously published results showing that high concentrations of trametinib were ineffective in A2058 melanoma^32^. We hypothesize that the administration of higher concentrations of trametinib may be followed by activation of anti-apoptotic pathways as was reported earlier^33^. Adding 2-DG did not significantly change the growth rate of the tumor while adding dabrafenib decreased the success of the treatment (Fig. 8c). Interestingly, adding either 2-DG or dabrafenib to trametinib led to increased pAkt and pS6 levels (Supp. Fig. 6d), suggesting again that random addition of drugs (i.e., not based on personalized signatures) to the treatment may evoke different, sometimes undesired, signaling feedback responses.

These results point to the significantly higher efficiency of the PaSSS-predicted combinations relative to drug combinations used in clinics. Moreover, we demonstrated the selectivity of the individualized treatments. The predicted and very effective combination for one BRAF^V600E^ melanoma malignancy was significantly less effective for the other, and vice versa (Fig. 8). Our results underscore the need for personalized treatment for each melanoma patient.

Although the predicted combinations achieved an effect superior to the combination used in clinics, they did not flatten the tumor growth curves in all cases. This raises the possibility that certain subpopulations in the tumors were overlooked. This can result from the growth of subpopulations that were initially very small and were therefore undetected in bulk proteomics. Alternatively, such subpopulations may form during the course of treatment due to new unbalanced processes that are induced in response to environmental changes (e.g., communication between cancer cells and stroma, which cannot be detected in in-vitro assays)^34^. Taking several biopsies during treatment may resolve such expanded, initially undetected, cellular subpopulations and help to adjust the personalized treatment accordingly.

## Discussion

With the accelerated gain of knowledge in the field of melanoma therapy and cancer research, it is becoming clear that tumors evolving from the same anatomical origins cannot necessarily be treated the same way^35^. Inter tumor heterogeneity results in various response rates of patients to therapy^36–38^. Herein we extend this notion and show that even tumors that were initially driven by the same oncogenes, specifically BRAF^V600E^-driven melanoma tumors, often evolve in different molecular manners^39^, giving rise to distinct altered signaling signatures, or PaSSS (patient-specific altered signaling signature), at the time of biopsy. We show that 17 altered molecular processes are repetitive among the 725 SKCM and THCA tumors. Each tumor is characterized by a specific PaSSS, i.e., a subset of ~1–3 unbalanced processes. Accordingly, each patient is assigned a unique barcode, denoting this PaSSS. We show that the collection of 725 tumors is described by 138 *distinct* barcodes, suggesting that the cohort of patients consists of 138 types of cancer, rather than only 4 types (SKCM or THCA; BRAF^WT^ or BRAF^V600E^). These 138 types of tumors, each representing a barcode, or a sub-combination of 17 unbalanced processes, are mapped into a multi-dimensional space, consisting of 17 dimensions. Once the tumor-specific information is transformed into a multi-dimensional space, treating these thousands of tumors becomes at an arm’s reach. The specific barcode assigned to each patient allows the rational design of patient-tailored combinations of drugs, many of which already exist in clinics.

We found that 353 BRAF^V600E^ and BRAF^WT^ melanoma tumors are described by 87 distinct barcodes of unbalanced processes and that 372 BRAF^V600E^ and BRAF^WT^ THCA tumors are described by 54 barcodes. Interestingly, the barcodes appeared to be almost mutually exclusive between SKCM and THCA tumors (Supp. Data 4). While this finding suggests that the molecular processes underlying SKCM and THCA tumor evolution may have organ-specific differences, a large number of cancer type-specific barcodes and the large number of barcodes describing single patients underscore the need for personalized diagnosis and treatment.

We show that tumors harboring BRAF^V600E^ can harbor distinct PaSSSs, and in contrast, that tumors can harbor the same PaSSS regardless of whether they carry BRAF^V600E^ or BRAF^WT^. We therefore deduce that profiling melanoma patients according to their BRAF mutational status is insufficient to assign effective therapy to the patient. Since the unbalanced processes each harbor a specific group of co-expressed altered proteins, they should all be targeted simultaneously to reduce the altered signaling flux in the tumor.

We demonstrate this concept experimentally by analyzing a cell line dataset and predicting efficiently targeted drug combinations for three selected BRAF^V600E^ melanoma cell lines, G361, A375, and A2058. We show that although all cell lines contain the mutated BRAF^V600E^, they harbor distinct barcodes, and demand different combinations of drugs (Figs. 5–8). We demonstrate that our PaSSS-based combinations were significantly more efficient than the drug combination often prescribed clinically to BRAF^V600E^ patients, dabrafenib+trametinib (Figs. 5–8). Moreover, we demonstrated the selectivity of the PaSSS-based drug combinations. The highly efficient PaSSS-based drug combination for one melanoma malignancy can be significantly less efficient for another melanoma and vice versa.

We note, however, that the PaSSS-based drug combinations did not achieve complete flattening of the tumor growth curves in vivo in some of the cases (Fig. 8). We hypothesize that an approach that, for example, tests the tumor several times during the treatment to examine whether small, previously undetected cellular subpopulations have expanded due to, for example, stroma-tumor communication, might be required^34^. A more holistic approach that combines immunotherapy might be beneficial as well. This is a highly interesting topic that is currently under study in our laboratory.

The results reported here highlight the urgent need for the design of personalized treatments for melanoma patients based on individualized alterations in signaling networks rather than on initial mutational events. Furthermore, the study establishes PaSSS analysis as an effective approach for the design of personalized cocktails comprising FDA-approved drugs. Personalized targeted cocktails, which may be further combined with immunotherapy strategies, are expected to provide long-term efficacy for melanoma patients.

## Methods

### Datasets

This study utilized a protein expression dataset consisting of 353 skin cutaneous melanoma (SKCM) samples and 372 thyroid carcinoma (THCA) samples. The samples were selected from a large TCPA dataset containing 7694 cancer tissues from various anatomical origins (PANCAN32, level 4 (The Cancer Proteome Atlas Portal, http://tcpaportal.org)). Each cancer tissue was profiled on a reverse-phase protein array (RPPA) for 258 cancer-associated proteins. After filtering out proteins that had NA values for a significant number of patients, 216 proteins remained for further analysis.

The dataset for the cancer cell lines was downloaded from the TCPA portal (The Cancer Proteome Atlas Portal, http://tcpaportal.org). The data was already published by Li et al.^40^. A part of the original dataset containing 290 cell lines from 16 types of cancers was selected, including breast, melanoma, ovarian, brain, blood, lung, colon, head and neck, kidney, liver, pancreas, bone, and different types of sarcomas, stomach-esophagus, uterus and thyroid cancers. The cell lines in the dataset were profiled for 224 phospho-proteins and total proteins using RPPA.

### Surprisal analysis

Surprisal analysis is a thermodynamic-based information-theoretic approach^41–43^. The analysis is based on the premise that biological systems reach a balanced state when the system is free of constraints^44–46^. However, when under the influence of environmental and genomic constraints, the system is prevented from reaching the state of minimal free energy, and instead reaches a state which is higher in free energy (in biological systems, which are normally under constant temperature and constant pressure, minimal free energy equals maximal entropy).

Surprisal analysis can take as input the expression levels of various macromolecules, e.g., genes, transcripts, or proteins. However, be it environmental or genomic alterations, it is the proteins that constitute the functional output in living systems, therefore we base our analysis on proteomic data. The varying forces or constraints, that act upon living cells ultimately manifest as alterations in the cellular protein network. Each constraint induces a change in a specific part of the protein network in the cells. The subnetwork that is altered due to the specific constraint is termed an unbalanced process. The system can be influenced by several constraints thus leading to the emergence of several unbalanced processes. When tumor systems are characterized, the specific set of unbalanced processes is what constitutes the tumor-specific signaling signature.

Surprisal analysis discovers the complete set of constraints operating on the system in any given tumor, *k*, by utilizing the following equation:^47^ ln $X_i\left( k \right) = \ln X_i^0\left( k \right)-{\Sigma}G_{i\alpha }\lambda _\alpha \left( k \right)$, where *i* is the protein of interest, $X_i^0$ is the expected expression level of the protein when the system is at the steady-state and free of constraints, and ${\Sigma}G_{i\alpha }\lambda _\alpha (k)$ represents the sum of deviations in the expression level of the protein *i* due to the various constraints, or unbalanced processes, that exist in the tumor *k*.

The term *G*_*iα*_ denotes the degree of participation of protein *i* in the unbalanced process α, and its sign indicates the correlation or anti-correlation between proteins in the same process (Supp. Data 1). Proteins with significant *G*_*iα*_ values are grouped into unbalanced processes (Supp. Fig. 1, Supp. Data 2) that are active in the dataset^10^.

The term *λ*_*α*_*(k)* represents the importance of the unbalanced process α in the tumor *k* (Supp. Data 1).

The partial deviations in the expression level of the protein *i* due to the different constraints sum up to the total change in expression level (relative to the balance state level), ${\Sigma}G_{i\alpha }\lambda _\alpha (k)$.

For complete details regarding the analysis please refer to the SI of reference^11,47^.

### Determination of the number of significant unbalanced processes

The analysis of the 725 patients provided a 725 × 216 matrix of *λ*_*α*_*(k)* values, such that every row in the matrix contained 216 values of *λ*_*α*_*(k)* for 725 patients, and each row corresponding to an unbalanced process (Supp. Data 1). However, not all unbalanced processes are significant. Our goal is to determine how many unbalanced processes are needed to reconstruct the experimental data, i.e., for which value of *n*: ln (*X*_*i*_*(k)/M)* ≈ *−ΣG*_*iα*_*λ*_*α*_*(k)*. To find *n*, we performed the following two steps:

#### Reproduction of the experimental data by the unbalanced processes was verified

We plotted *ΣG*_*iα*_*λ*_*α*_*(k)* for *α* = 1, 2, …, *n* against ln *X*_*i*_*(k)* for different proteins, *i*, and for different values of *n*, and examined the correlation between them as *n* was increased. An unbalanced process, *α* = *n*, was considered significant if it improved the correlation significantly relative to *α* = *n* *–* 1 (Supp. Fig. 2) (see reference ^9^ for more details).

#### Processes with significant amplitudes were selected

To calculate threshold limits for *λ*_*α*_*(k)* values (presented in Supp. Data 1 and Supp. Fig. 3) the standard deviations of the levels of the 10 most stable proteins in this dataset were calculated (e.g., those with the smallest standard deviations values). Those fluctuations were considered as baseline fluctuations in the population of the patients which are not influenced by the unbalanced processes. Using standard deviation values of these proteins the threshold limits were calculated as described previously^48^. The analysis revealed that from *α* = 18, the importance values, *λ*_*α*_*(k)*, become insignificant (i.e., do not exceed the noise threshold), suggesting that 17 unbalanced processes are enough to describe the system.

For more details see references^10,47^.

### Generation of functional subnetworks

The functional sub-networks presented in Figs. 2, 5, 6, and Supp. Figs. 1 and 5 were generated using a python script as described previously^10^. Briefly, the goal was to generate a functional network according to the STRING database, where proteins with negative G values are marked blue and proteins with positive G values are marked red, to easily identify the correlations and anti-correlations between the proteins in the network. The script takes as an input the names of the genes in the network and their *G* values, obtain the functional connections and their weights from the STRING database (string-db.org), and then plots the functional network (using matplotlib library).

### Barcode calculation

The barcodes of unbalanced processes were generated using a python script. For each patient, *λ*_*α*_*(k)* (*α* = 1, 2, 3, …, 17) values were normalized as follows: If *λ*_*α*_*(k)* *>* *2* (and is, therefore, significant according to the calculation of threshold values) then it was normalized to 1; if *λ*_*α*_*(k)* *<* *−2* (significant according to threshold values as well) then it was normalized to −1; and if *−2* < *λ*_*α*_*(k)* *<2* then it was normalized to 0.

### Cell culture

The BRAF mutated melanoma cell lines, A375, G361, and A2058 were obtained from the ATCC and grown in DMEM (G361 and A2058) or RPMI (A375) medium. The cells were supplemented with 10% fetal calf serum (FCS), L-glutamine (2 mM), 100 U/ml penicillin, and 100 mg/ml streptomycin and incubated at 37֯ °C in 5% CO_2_. The cell lines were authenticated at the Biomedical Core Facility of the Technion, Haifa, Israel.

### Antibodies and western blot analysis

The cells were seeded into 6 well plates (~1.5 × 10^6^ cells/well) and grown under complete growth media. A375 cells were treated the next day as indicated for 48 h in a partial starvation medium (RPMI medium with 1.2% FCS). G361 and A2058 cells were treated in a complete growth medium for 24 h. The dead cells were collected from the medium. The adherent cells were then treated with IGF for 15 min. The cells were then lysed using hot sample buffer (10% glycerol, 50 mmol/L Tris-HCl pH 6.8, 2% SDS, and 5% 2-mercaptoethanol) and western blot analysis was carried out. The lysates were fractionated by SDS-PAGE and transferred to nitrocellulose membranes using a transfer apparatus according to the manufacturer’s protocols (Bio-Rad). Blots were developed with an ECL system according to the manufacturer’s protocols (Bio-Rad).

Anti-p-PKM2 (Tyr105) (cat. no. 3827S; 1:1000), anti-p-S6 (Ser235/236) (cat. no. 4858S; 1:1000), anti-p-PDGFRβ (Y751) (cat. no. 4549S; 1:1000), anti-p-AKT (Ser473) (cat. no. 4060S; 1:1000), anti-p-p70S6K (Tyr389) (cat. no. 9205L; 1:1000), anti-p-Mek (Ser217/221) (cat. no. 9154S; 1:1000) and anti-total –PARP (cat. no. 9542S; 1:1000) antibodies were purchased from Cell Signaling Technology, Inc. Anti-p-ERK2 (E4) (cat. no. SC7383; 1:200), anti-total-P53 (cat. no. SC126; 1:200) and anti-total-GAPDH (cat. no. SC47724; 1:200) antibodies were purchased from Santa Cruz Biotechnology.

In each of the figures, all blots were derived from the same experiment and were processed in parallel.

### Methylene blue assay

In a 96 well plate, the cells were seeded and treated as indicated for 72 h. The cells were fixed with 4% paraformaldehyde and then stained with methylene blue. To calculate the number of surviving cells, the color was extracted by adding 0.1 M Hydrochloric acid and the absorbance was read at 630 nm.

### MTT assay

Cells were seeded and treated as indicated in a 96 well plate for 72 h. Cell viability was checked using an MTT assay kit (Abcam). Equal volumes of MTT solution and culture media were added to each well and incubated for 3 h at 37֯ °C. MTT solvent was added to each well, and then the plate was covered in aluminum foil and put on the orbital shaker for 15 min. Absorbance was read at 590 nm within 1 h.

### Resistance assay

Cells were seeded in multiple 96 well plates and treated as needed in various time points (3, 7, 14, 21, 28 days). At every time point, the cells were fixed with 4% paraformaldehyde and then stained with methylene blue. The number of cells that survived at each time point was quantified by adding 0.1 M Hydrochloric acid and reading the absorbance at 630 nm.

### Animal studies

The cells—A375 (0.25 × 10^6^ cells/mouse), G361 (0.5 × 10^6^ cells/mouse), or A2058 (0.5 × 10^6^ cells/mouse)—were inoculated subcutaneously into NSG mice (*n* = 7–8 mice per group), and once the volume of the tumors reached 50 mm^3^, treatments were initiated 6 times a week for up to 4 weeks. Tumor volume was measured twice a week. Trametinib (0.5 mg/kg), dasatinib (35 mg/kg) and dabrafenib (35 mg/kg) were suspended in an aqueous mixture of 0.5% hydroxypropyl methylcellulose + 0.2% tween 80 and administered by oral gavage. 2-deoxy-D-glucose (500 mg/kg) was suspended in saline and injected intraperitoneally. All the drugs were purchased from Cayman chemicals (Enco, Israel). The Hebrew University is an AAALAC International accredited institute. All experiments were conducted with approval from the Hebrew University Animal Care and Use Committee. Ethical accreditation number: Md-17-15174-4.

### Reporting summary

Further information on research design is available in the Nature Research Reporting Summary linked to this article.

## Supplementary information

Supplementary Data

Supplementary Data 1

Supplementary Data 2

Supplementary Data 3

Supplementary Data 4

Supplementary Data 5

Supplementary Data 6

Supplementary Data 7

Supplementary Data 8

Supplementary Data 9

Reporting Summary

## Data Availability

The human tumor dataset that supports the findings of this study is publicly available for download from the TCPA portal (The Cancer Proteome Atlas Portal, http://tcpaportal.org), https://tcpaportal.org/tcpa/download.html Pan-Can 32. The cell line dataset that supports the findings of this study is publicly available for download from the TCPA portal (The Cancer Proteome Atlas Portal, http://tcpaportal.org), https://tcpaportal.org/mclp/#/datasets.
