# Supplementary material for: Overcoming resistance to BRAFV600E inhibition in melanoma by deciphering and targeting personalized protein network alterations
Source: NPJ Precis Oncol. 2021 Jun 10;5:50. doi: 10.1038/s41698-021-00190-3 (PMC8192524; doi:10.1038/s41698-021-00190-3)
Supplement: Supplementary file 1 — Supplementary Data [file 41698_2021_190_MOESM1_ESM.pdf]

Supplementary Information

Overcoming BRAF^{V600E} Acquired Resistance in Melanoma by Deciphering and Targeting Personalized Protein Network Alterations

S. Vasudevan#, E. Flashner-Abramson#, Heba Alkhatib, Sangita Roy Chowdhury, I. Adesoji Adejumobi, D. Vilencki, S. Stefansky, A.M. Rubinstein and N. Kravchenko-Balasha*

The Institute of Biomedical and Oral Research, Hebrew University of Jerusalem, Jerusalem 91120, Israel

*Corresponding author: natalyk@ekmd.huji.ac.il

# Equal contribution

Table of contents:

Contents

Supplementary Figure 1. 2

Supplementary Figure 2. 5

Supplementary Figure 3. 6

Supplementary Figure 4. 9

Supplementary Figure 5. 10

Supplementary Figure 6. 13

Supplementary Figure 7. 14

Supplementary Data Legends 15

Original Western Blots 16

Supplementary References 23

Supplementary Figures

Supplementary Figure 1.

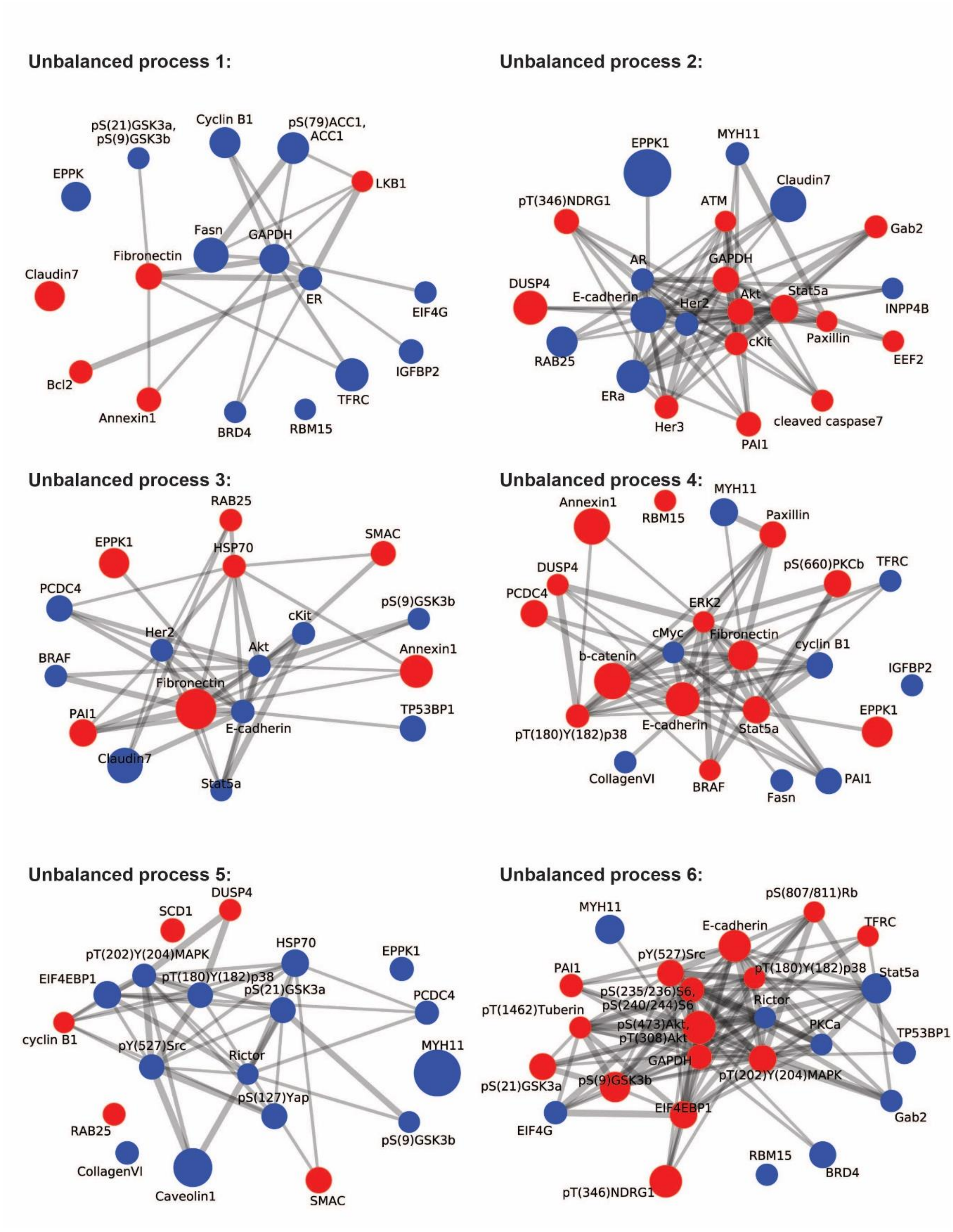

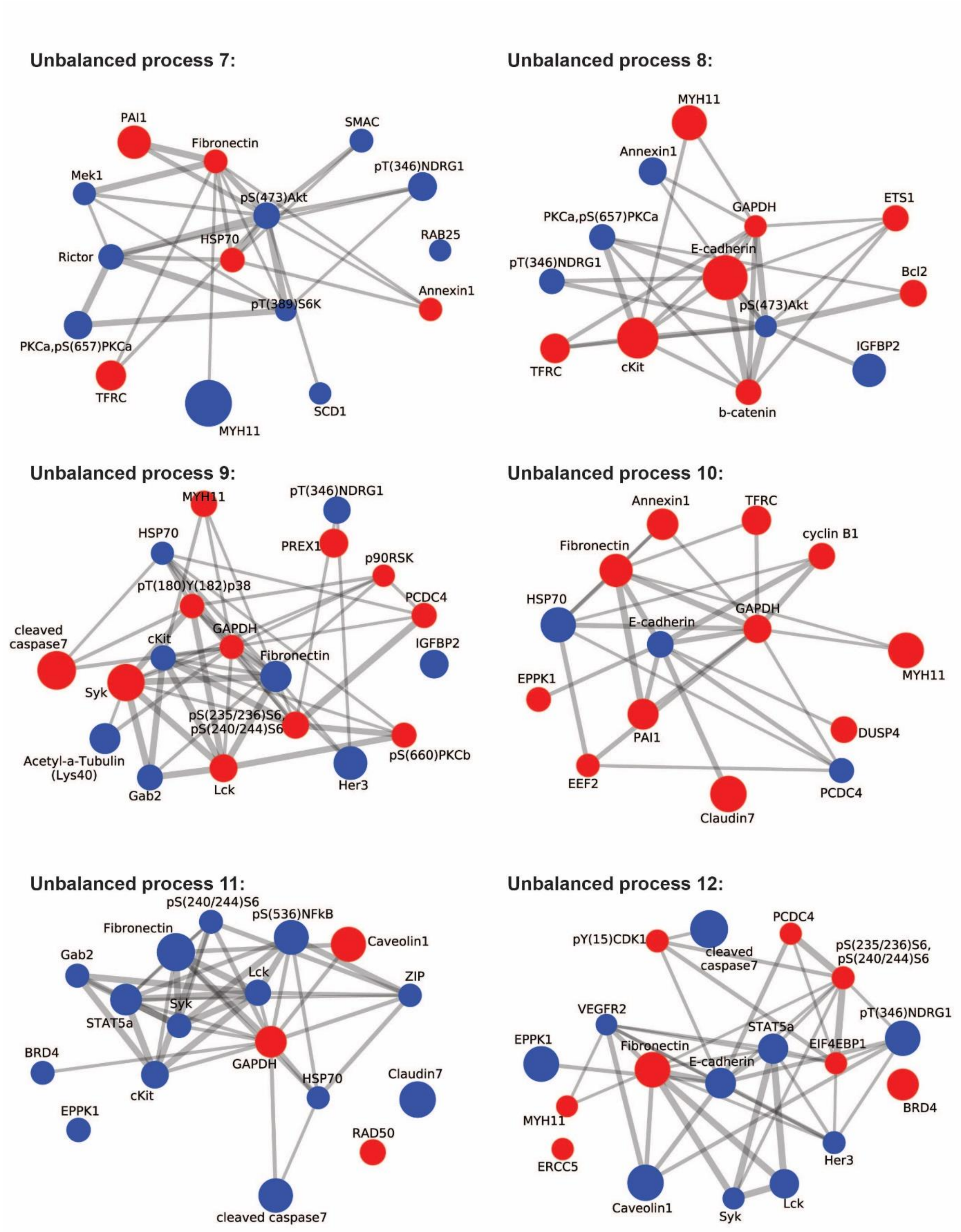

Unbalanced process 7:
PAI1
Fibronectin
SMAC
Mek1
pT(346)NDRG1
pS(473)Akt
HSP70
RAB25
Rictor
pT(389)S6K
Annexin1
PKCa,pS(657)PKCa
TFRC
SCD1
MYH11
Unbalanced process 8:
MYH11
Annexin1
ETS1
GAPDH
PKCa,pS(657)PKCa
E-cadherin
pT(346)NDRG1
Bcl2
pS(473)Akt
IGFBP2
TFRC
cKit
b-catenin
Unbalanced process 9:
MYH11
pT(346)NDRG1
HSP70
PREX1
p90RSK
pT(180)Y(182)p38
PCDC4
cleaved
caspase7
GAPDH
cKit
IGFBP2
Fibronectin
Syk
pS(235/236)S6,
pS(240/244)S6
Acetyl-a-Tubulin
(Lys40)
pS(660)PKCb
Gab2
Lck
Her3
Unbalanced process 10:
Annexin1
TFRC
cyclin B1
Fibronectin
HSP70
GAPDH
E-cadherin
MYH11
EPPK1
PAI1
DUSP4
EEF2
PCDC4
Claudin7
Unbalanced process 11:
pS(240/244)S6
pS(536)NFkB
Fibronectin
Caveolin1
Gab2
Lck
ZIP
Syk
STAT5a
BRD4
GAPDH
HSP70
Claudin7
cKit
EPPK1
RAD50
cleaved caspase7
Unbalanced process 12:
PCDC4
pY(15)CDK1
cleaved
caspase7
pS(235/236)S6,
pS(240/244)S6
VEGFR2
pT(346)NDRG1
STAT5a
EPPK1
Fibronectin
EIF4EBP1
E-cadherin
BRD4
MYH11
ERCC5
Her3
Caveolin1
Lck
Syk

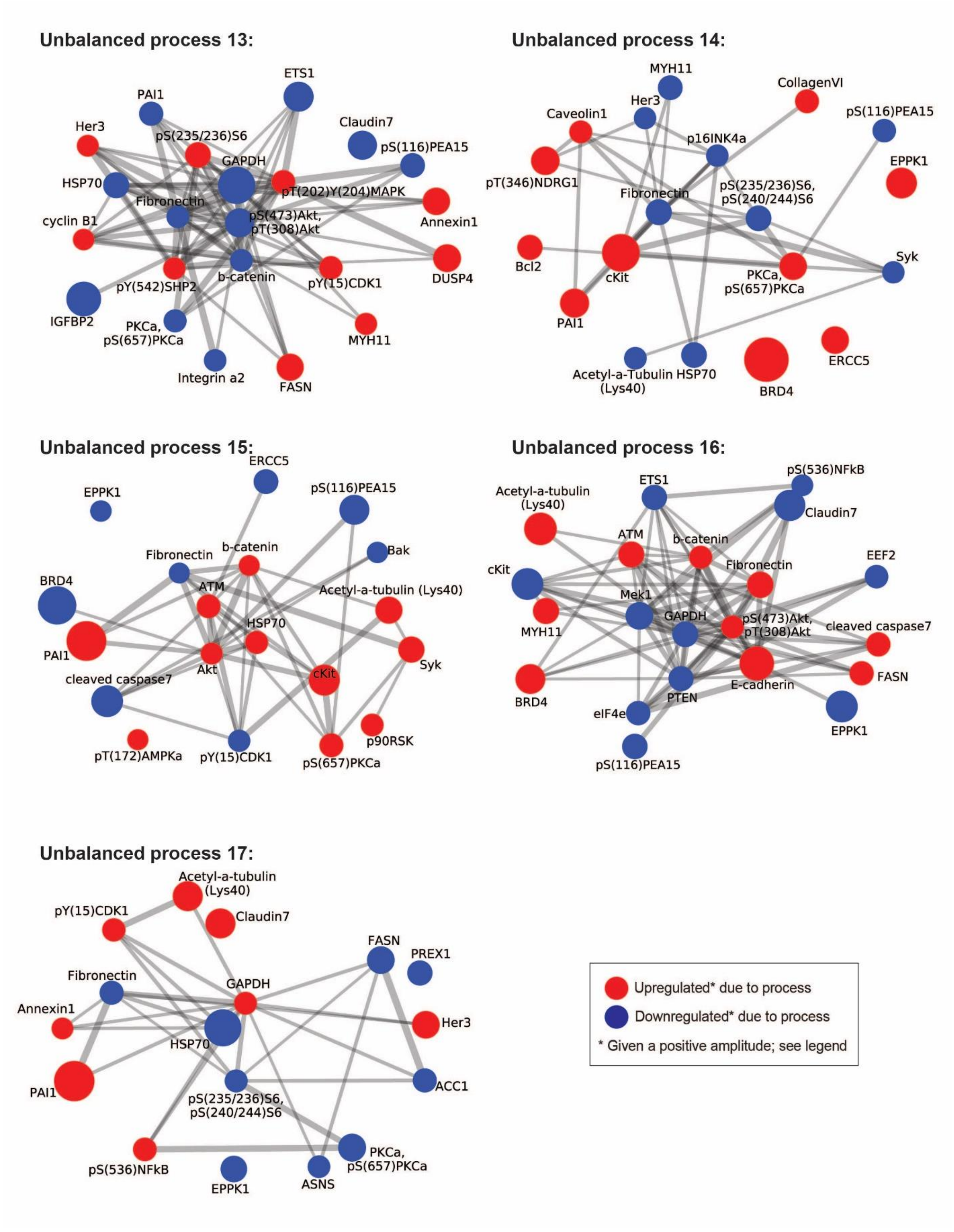

Supplementary Figure 1. The unbalanced subnetworks identified by PaSSS analysis for 725 SKCM and THCA tumors. For every process α, the proteins were assembled into networks using functional interactions according to STRING database. **<u>Note</u>** that red proteins are upregulated, and blue proteins are downregulated <u>given that the amplitude of the process is positive</u>. In tumors where the amplitude is negative, the direction of change is opposite.

Supplementary Figure 2.

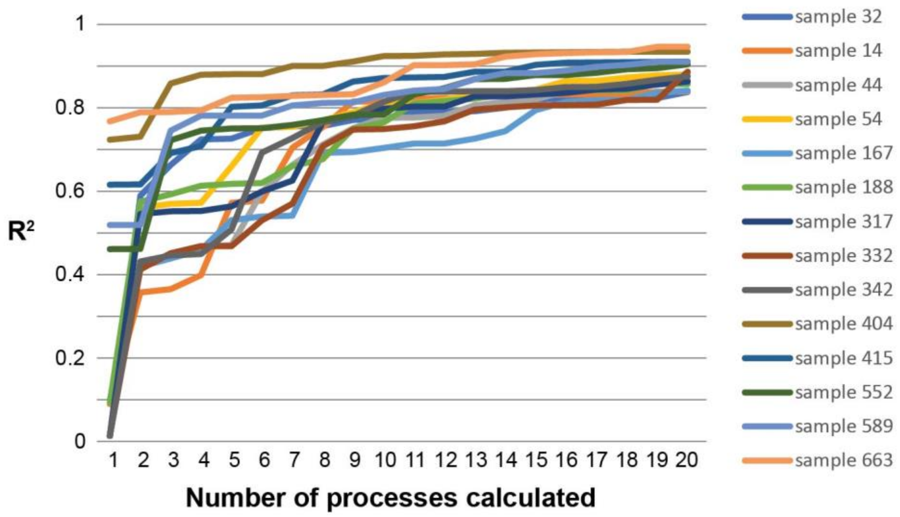

Supplementary Figure 2. 17 unbalanced processes suffice to describe the biological imbalance in 725 tumors. Presented are R^2 values for 14 random patients. The R^2 was calculated for every patient by plotting the natural logarithm of the experimental data (LnXi) vs. $\sum G_{i\alpha}\lambda_{\alpha}(k)$ for $\alpha = 0, 1, 2, ..., 20$. As more constraints are added, we expect to get higher agreement between the experimental data and the theoretical calculation, reaching the highest level of correlation when the experimental data is fully reproduced by the theoretical fitting. The plot reaches a plateau after the 17th constraint. This indicates that for $\alpha = 18, 19, 20...$ the data includes mainly noise.

Supplementary Figure 3.

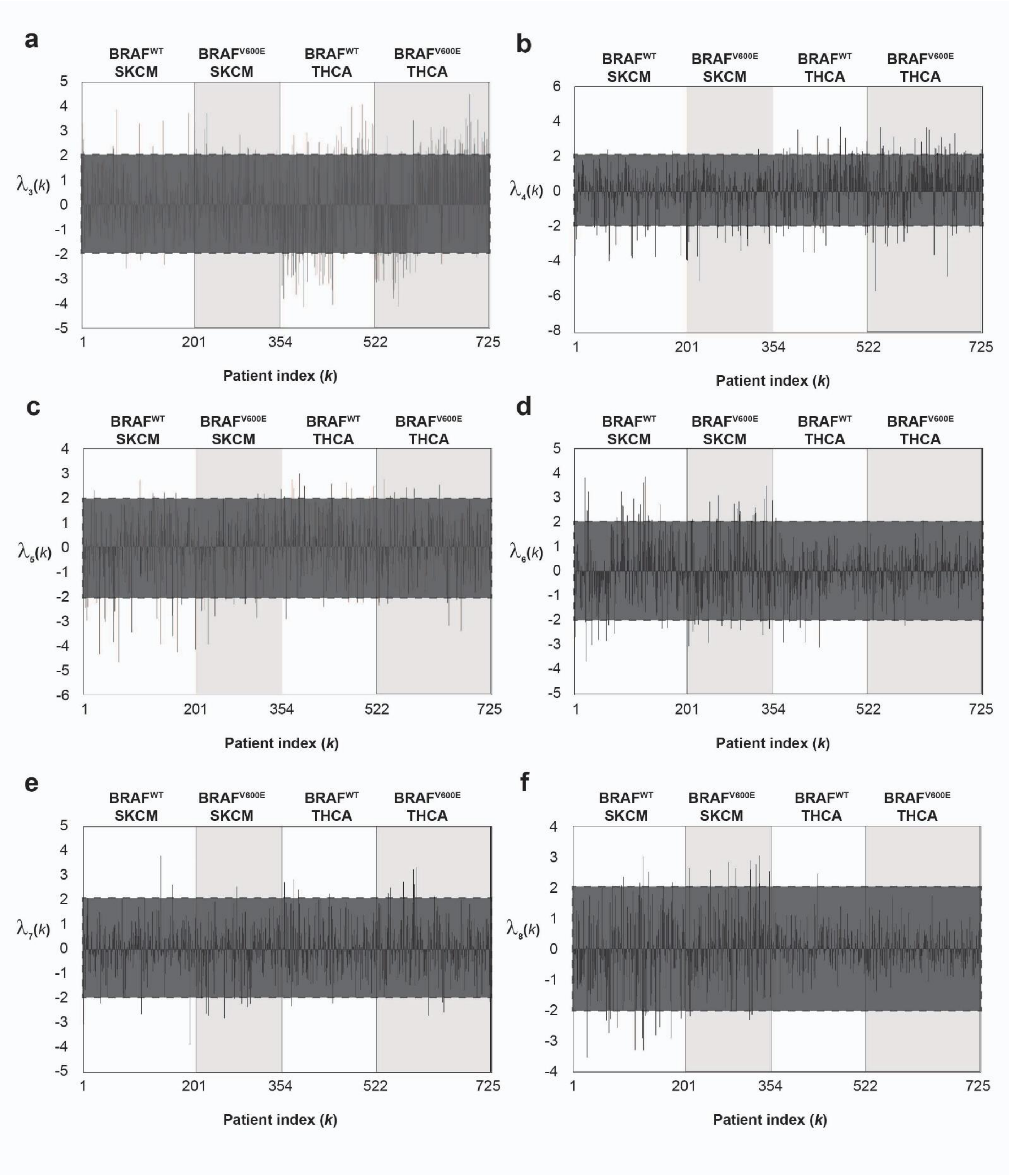

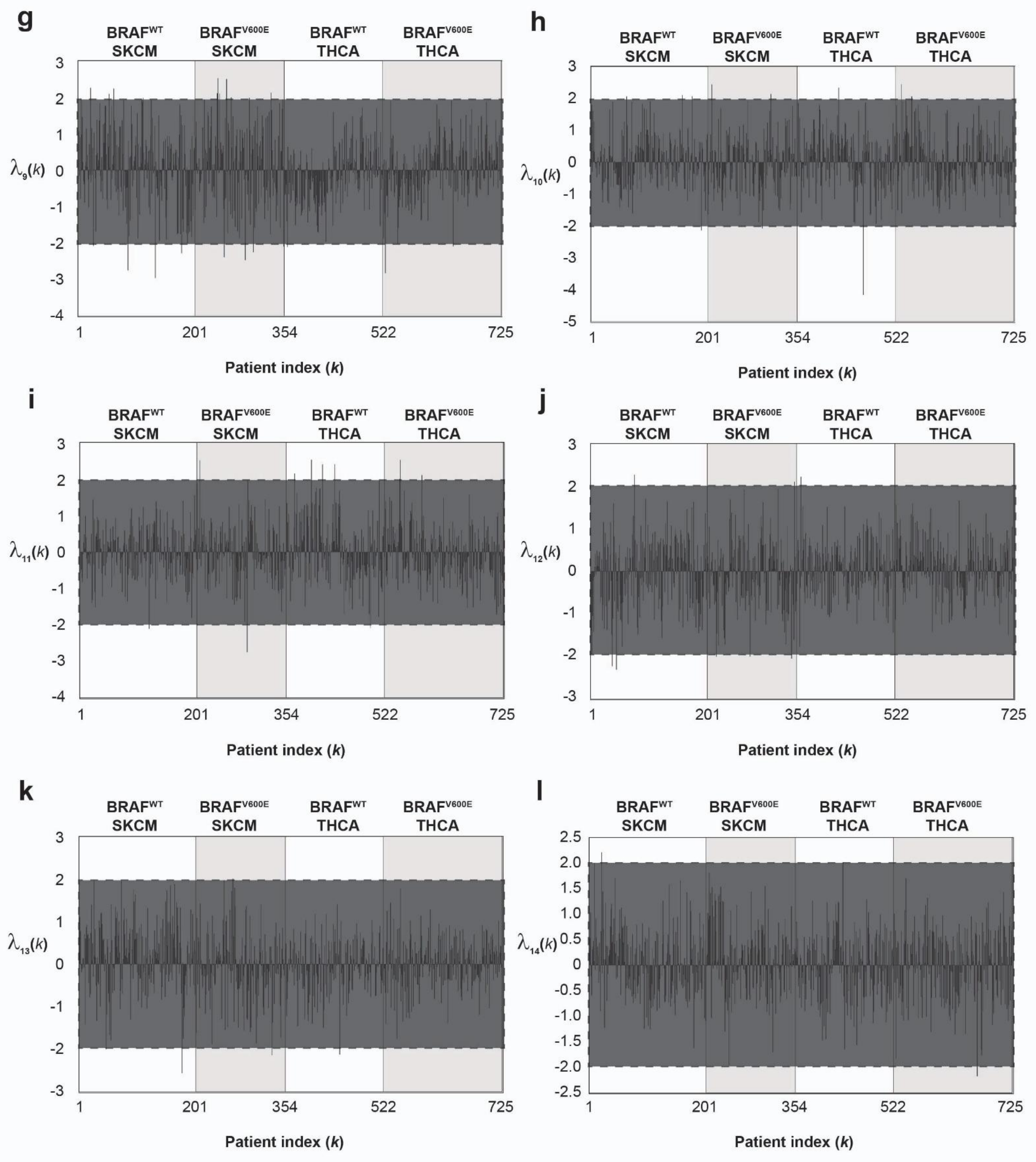
g
BRAF^WT SKCM
BRAF^V600E SKCM
BRAF^WT THCA
BRAF^V600E THCA
λ9(k)
Patient index (k)
h
λ10(k)
i
λ11(k)
j
λ12(k)
k
λ13(k)
l
λ14(k)
1
201
354
522
725

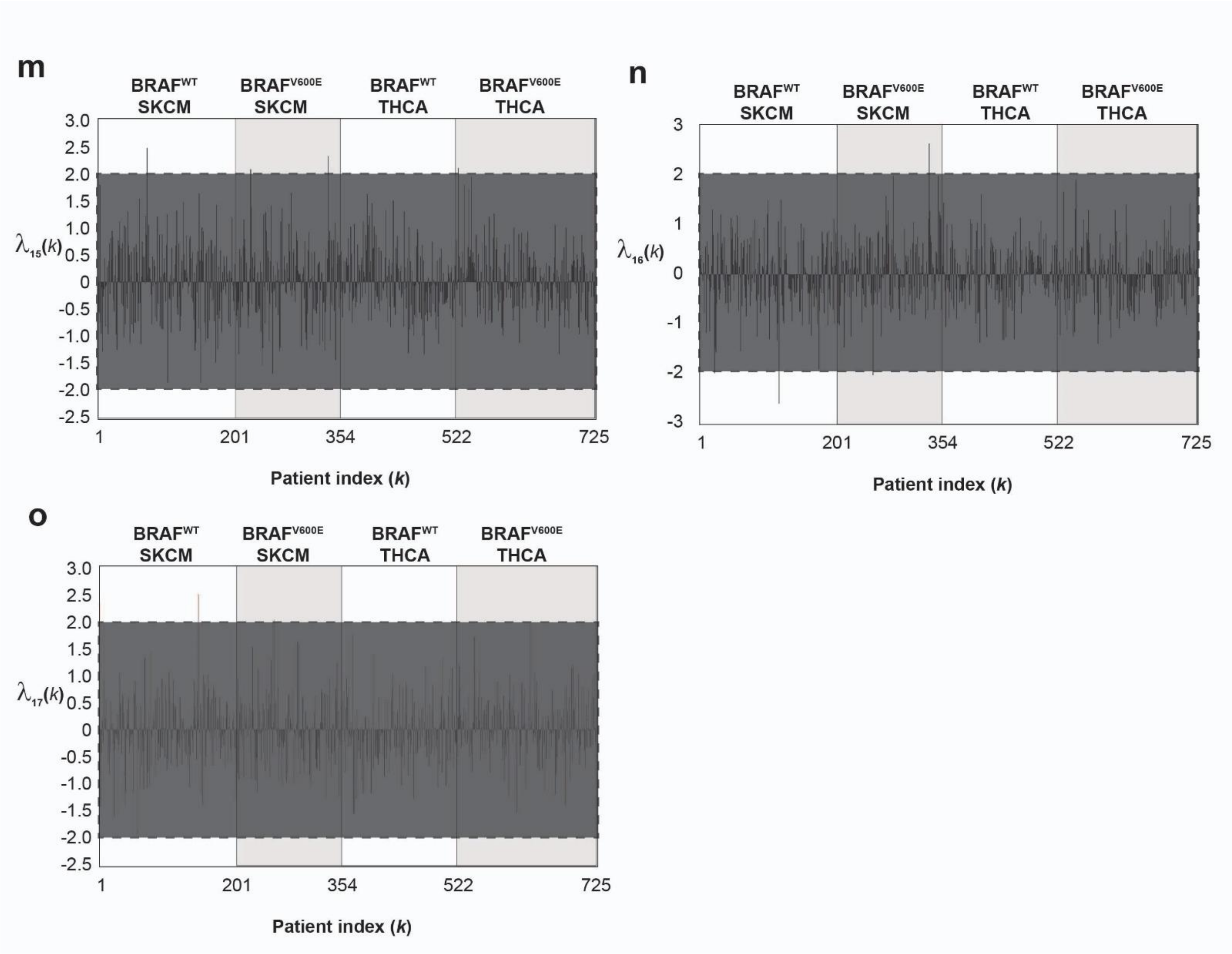

Supplementary Figure 3. Tumor-specific amplitudes (λα(k)) of the 17 unbalanced processes. Not all unbalanced processes are active in all patients. Rather, each unbalanced process, α, is assigned an amplitude, λα(k), for the specific tumor, k (see also Supplementary Data 1). Every panel in the figure presents 725 values of λα(k) for a specific value of α. The gray boxes mark the threshold limits – only values greater than 2 or smaller than -2 were considered significant (see Methods for more details).

Supplementary Figure 4.

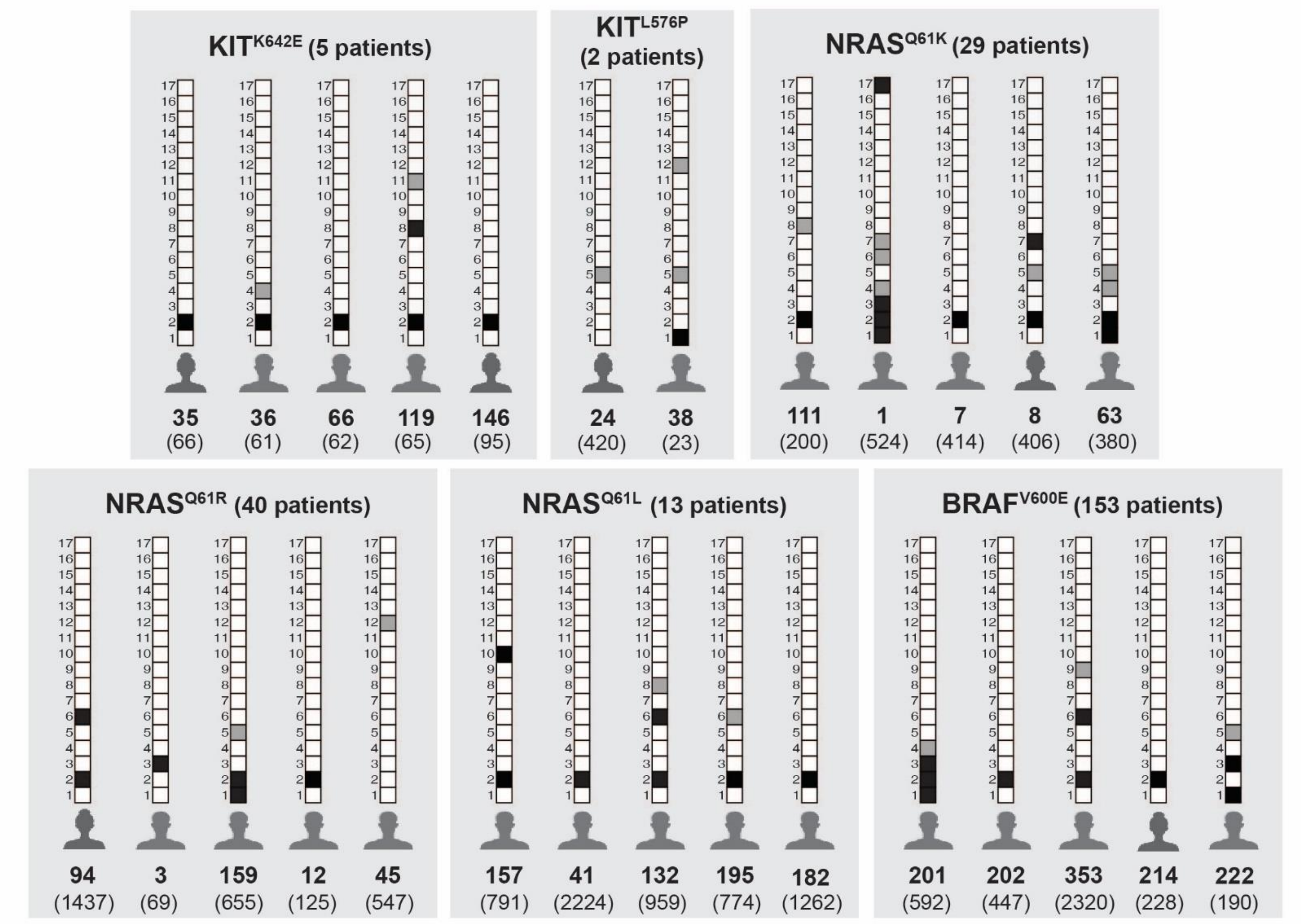

Supplementary Figure 4. SKCM patients harboring the same genomic mutations are characterized by various PaSSS-based barcodes. Patient-specific data regarding common mutations in melanoma was obtained from the GDC Data Portal. The full list can be found in Supplementary Data 6. For each mutation, the number of total patients harboring this mutation is stated. The mutations shown are mutually exclusive, namely a patient that harbors one of the mutations does not harbor any of the others [1]. Up to 5 patients were randomly selected for each mutation, and their PaSSS-based barcodes are shown, demonstrating that even though they carry the same mutation, their barcodes of unbalanced processes differ, and may thus demand distinct modes of treatment. The number in brackets (under patient index) denotes the total number of mutations, according to patient-specific data from cBioportal.org.

Supplementary Figure 5.

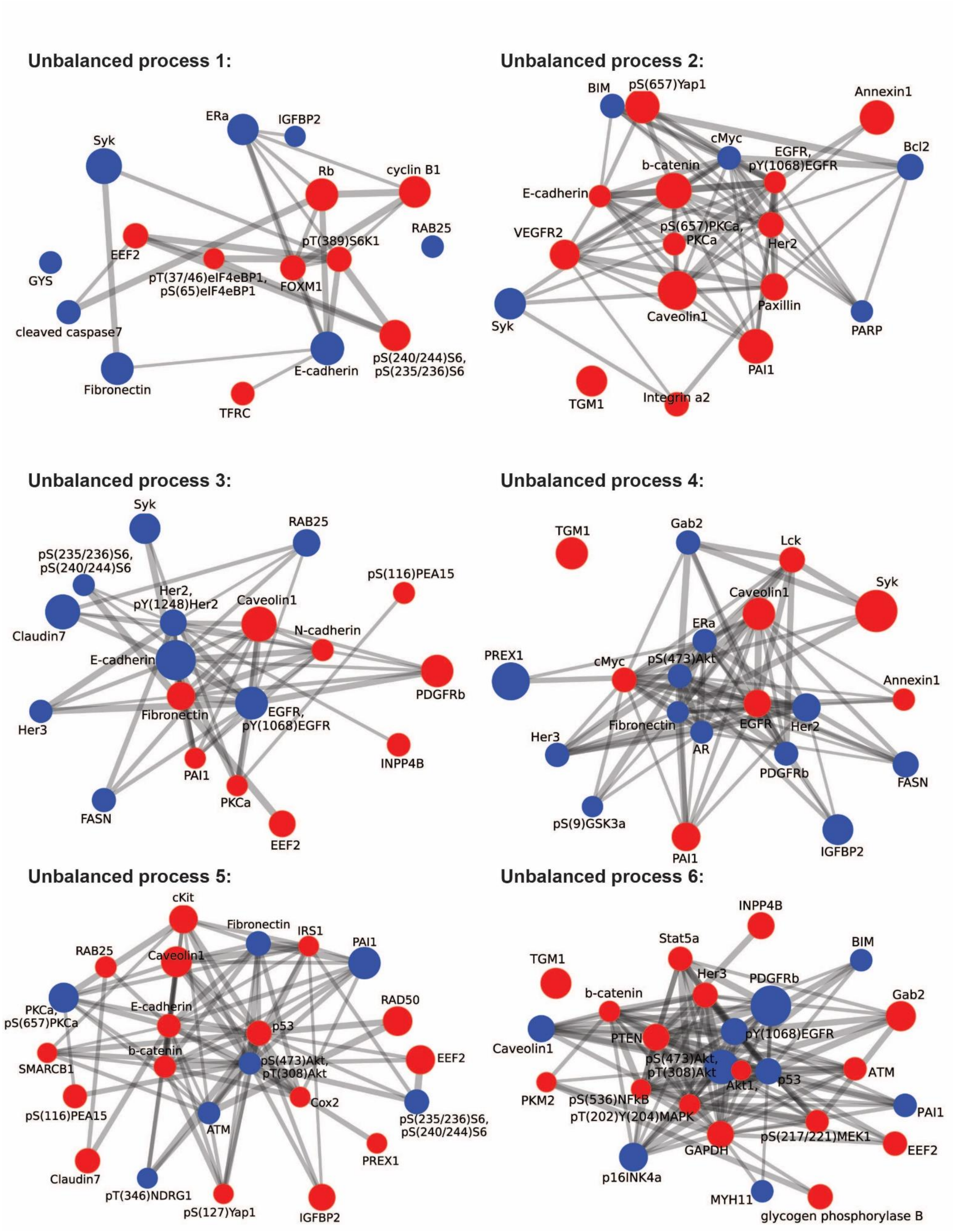

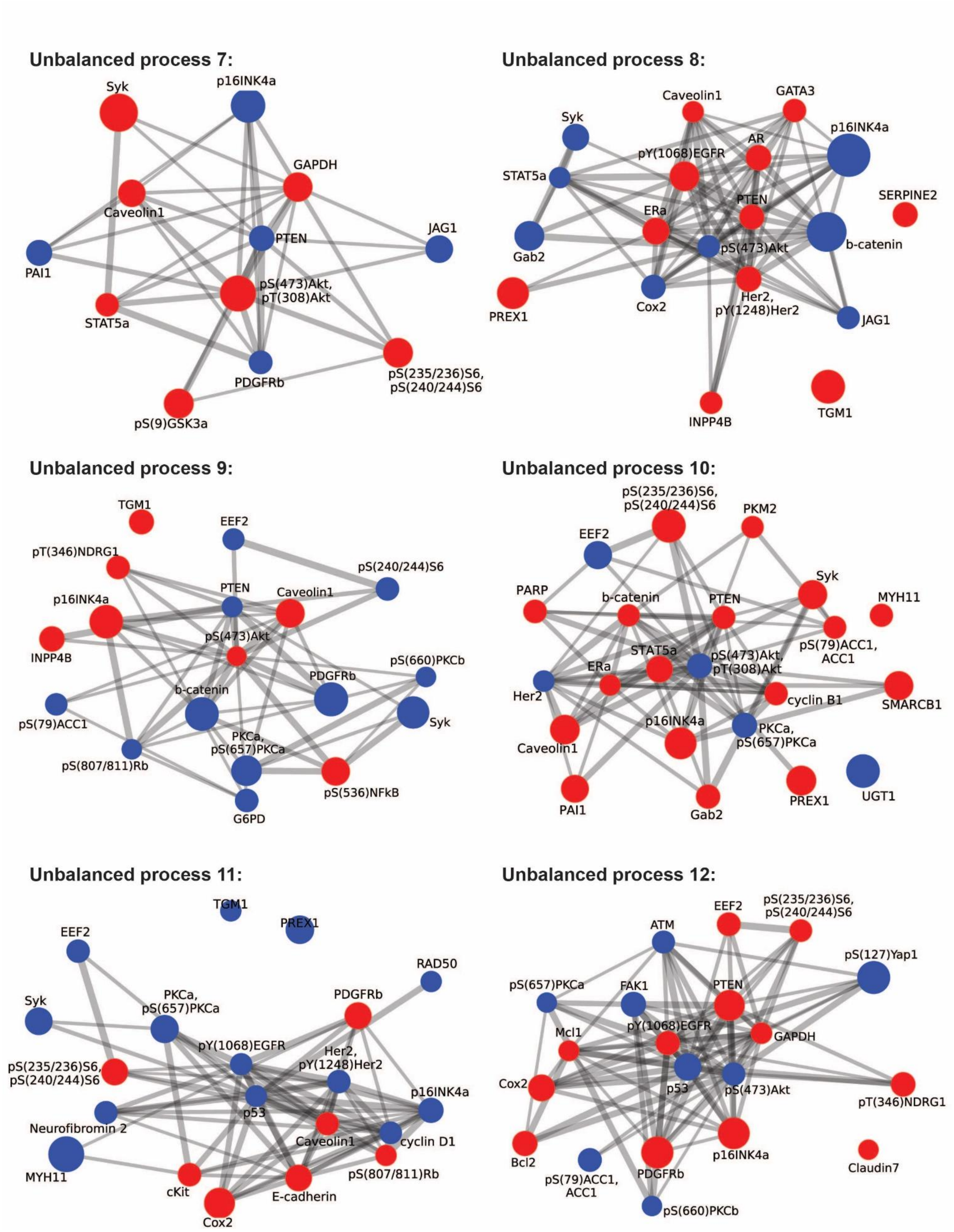
Unbalanced process 7:
Syk
p16INK4a
GAPDH
Caveolin1
JAG1
PTEN
PAI1
pS(473)Akt,
pT(308)Akt
STAT5a
PDGFRb
pS(235/236)S6,
pS(240/244)S6
pS(9)GSK3a
Unbalanced process 8:
Caveolin1
GATA3
Syk
p16INK4a
AR
pY(1068)EGFR
STAT5a
SERPINE2
PTEN
ERa
Gab2
pS(473)Akt
b-catenin
PREX1
Cox2
Her2,
pY(1248)Her2
JAG1
INPP4B
TGM1
Unbalanced process 9:
TGM1
EEF2
pT(346)NDRG1
pS(240/244)S6
PTEN
Caveolin1
p16INK4a
pS(473)Akt
INPP4B
pS(660)PKCb
PDGFRb
b-catenin
pS(79)ACC1
Syk
PKCa,
pS(657)PKCa
pS(807/811)Rb
pS(536)NFkB
G6PD
Unbalanced process 10:
pS(235/236)S6,
pS(240/244)S6
PKM2
EEF2
Syk
PARP
b-catenin
PTEN
MYH11
pS(79)ACC1,
ACC1
STAT5a
pS(473)Akt,
pT(308)Akt
ERa
Her2
cyclin B1
SMARCB1
p16INK4a
PKCa,
pS(657)PKCa
Caveolin1
PAI1
Gab2
PREX1
UGT1
Unbalanced process 11:
TGM1
PREX1
EEF2
RAD50
Syk
PKCa,
pS(657)PKCa
PDGFRb
pY(1068)EGFR
Her2,
pY(1248)Her2
pS(235/236)S6,
pS(240/244)S6
p16INK4a
p53
Neurofibromin 2
Caveolin1
cyclin D1
MYH11
pS(807/811)Rb
cKit
E-cadherin
Cox2
Unbalanced process 12:
EEF2
pS(235/236)S6,
pS(240/244)S6
ATM
pS(127)Yap1
pS(657)PKCa
FAK1
PTEN
Mcl1
pY(1068)EGFR
GAPDH
Cox2
p53
pS(473)Akt
pT(346)NDRG1
Bcl2
p16INK4a
Claudin7
pS(79)ACC1,
ACC1
PDGFRb
pS(660)PKCb

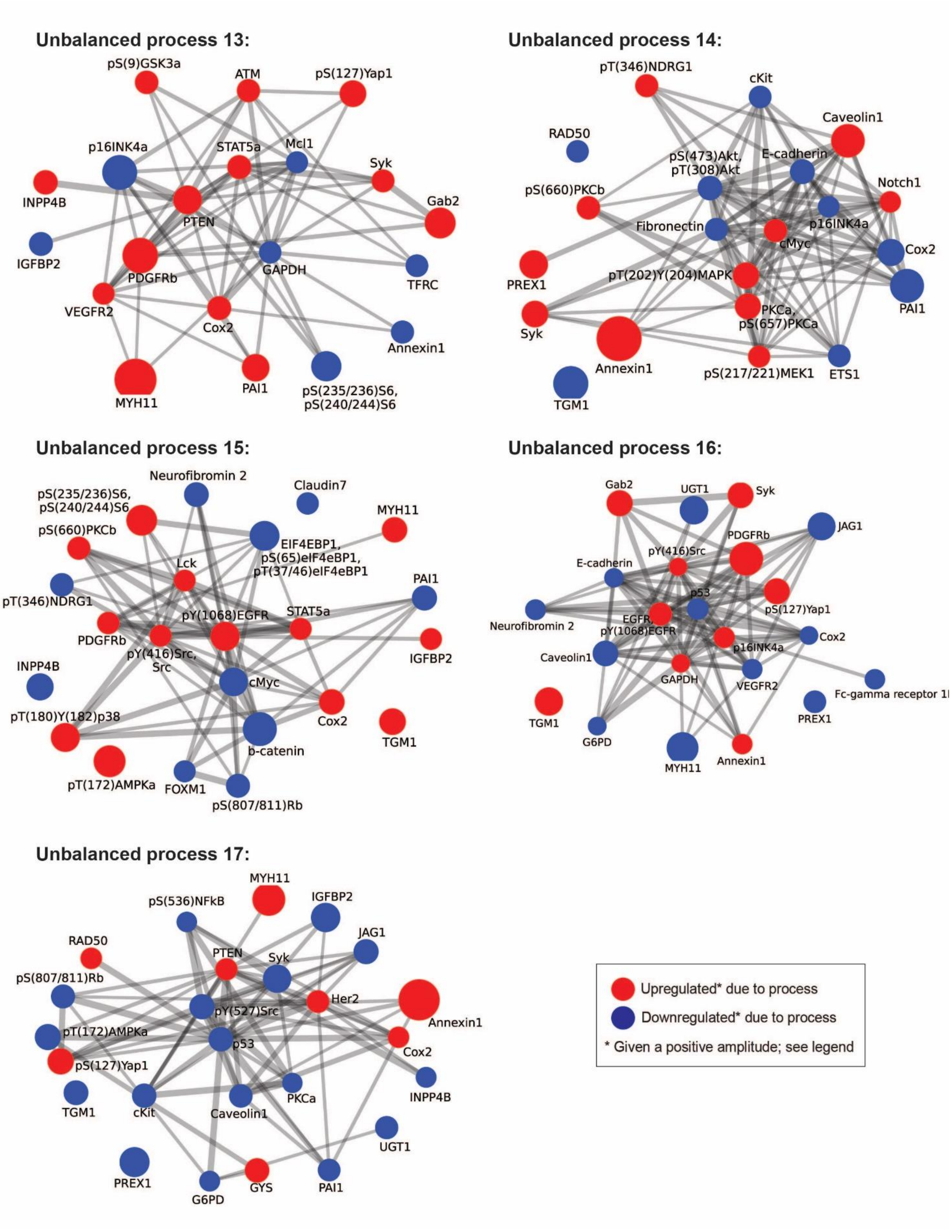

Supplementary Figure 5. The unbalanced subnetworks identified by PaSSS analysis for 219 cell lines. For every process α, the proteins were assembled into networks using functional interactions according to STRING database. **Note** that red proteins are upregulated, and blue proteins are downregulated given that the amplitude of the process is positive. In tumors where the amplitude is negative, the direction of change is opposite.

Supplementary Figure 6.

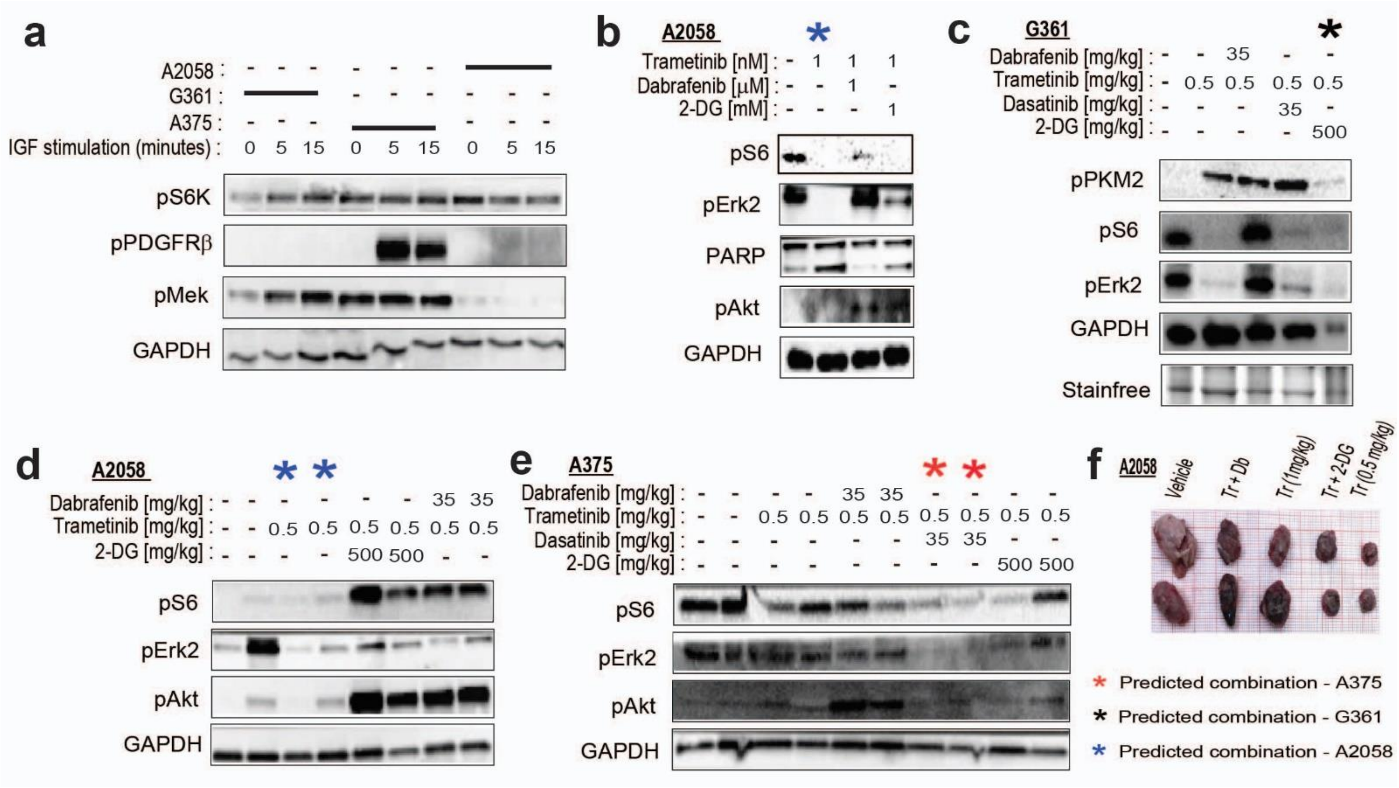

Supplementary Figure 6: Comparison of 3 BRAF-mutated melanoma malignancies and their altered signaling signatures in vitro and in vivo. (a) Western blot results comparing the basal state of 3 melanoma malignancies upon stimulation with IGF at different time points. The central druggable targets representing the PaSSS of each cell line are shown. pS6K (representative of unbalanced process 1) is expressed in all 3 cell lines. pPDGFR (representative of unbalanced process 3) is active only in A375 as suggested by the analysis, whereas pMEK (representative of unbalanced process 6) is active in A375 and G361. (**b**) Western blot results of A2058 following treatment with different therapies *in vitro*. The predicted therapy depletes the signaling in A2058 cells as represented by the decrease in phosphorylation levels of pS6 and pERK. (**c**) Western blot results of G361 tumors following treatment with different therapies *in vivo*. To show a long-term effect of the combined treatment on GAPDH levels, western blot analysis was performed on tissues harvested following 25 days of treatment. The predicted combination depletes the signaling in G361 tumors, as represented by the decrease in phosphorylation levels of pS6, pPKM2 and pERK. (**d**) Western blot results of A2058 tumors following treatment with different therapies *in vivo*. The predicted therapy depletes the signaling in A2058 tumors as represented by the decrease in phosphorylation levels of pAKT and pERK. (**e**) Western blot results of A375 tumors following treatment with different therapies *in vivo*. The effect of the predicted combinations on phospho-levels (d,e) was examined 7 days following the beginning of the treatment. (**f)** The size of A2058 tumors following treatment with different therapies. Lower concentrations of trametinib were more efficient in inhibiting tumor growth than higher concentrations.

Supplementary Figure 7.

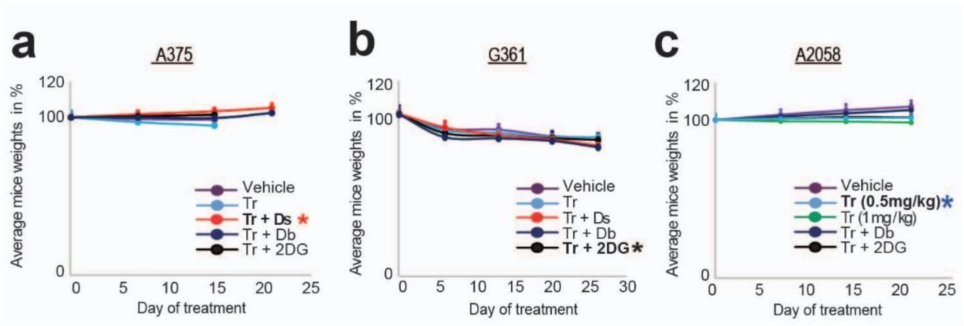

Supplementary Figure 7: Mice treated with SA-based drug combinations demonstrated no significant weight loss *in vivo*. A375 (**a**), G361 (**b**) or A2058 (**c**) were injected subcutaneously into mice, and once tumors reached 50 mm^3, treatments were initiated. In all three cases, the PaSSS-based drug combinations or the monotherapy of trametinib or the combinations predicted to partially target the PaSSS did not cause any significant weight loss in the mice (less than 12% from the start of treatment) during the course of the treatment.

Supplementary Data Legends

Supplementary Data 1-9 are provided as excel files.

Each Supplementary Data file includes a legend located in the first tab of the dataset. The legend provides full description of the content.

Original Western Blots

<u>Original blots for Figure 5:</u>

pS6

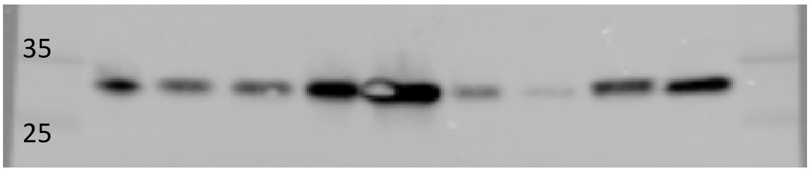

pErk2

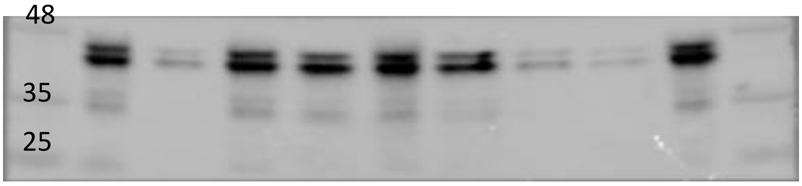

pAkt

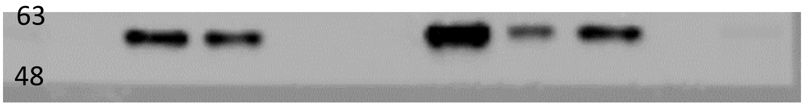

pPDGFRb

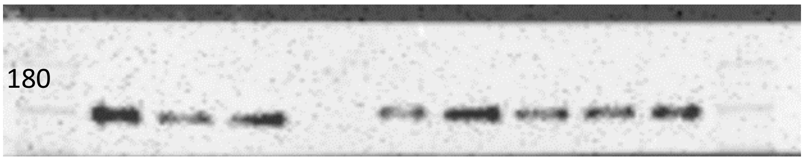

pPKM2

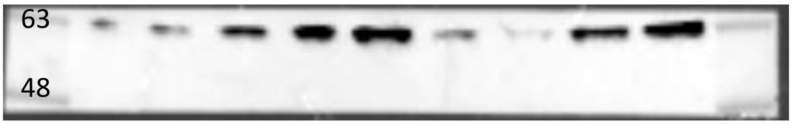

P53

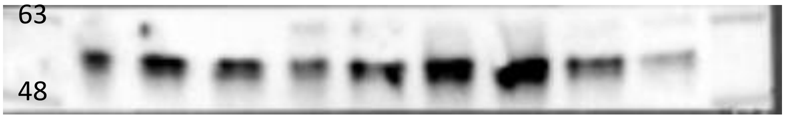

PARP

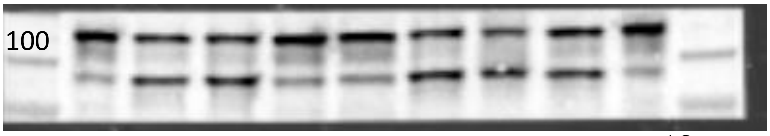

PS6K

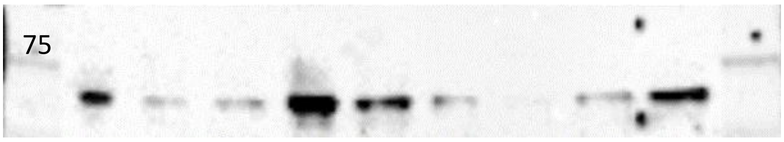

GAPDH

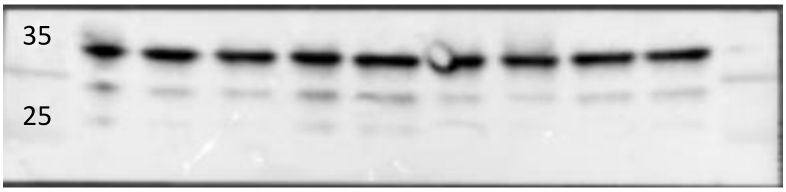

<u>Original blots for Figure 6:</u>

pS6

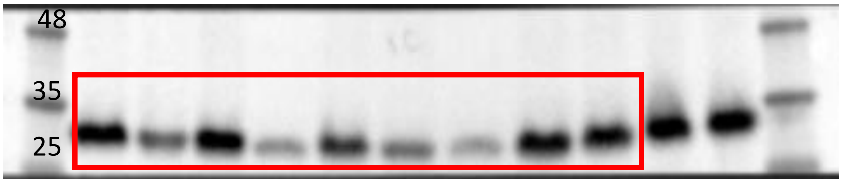

pERK2

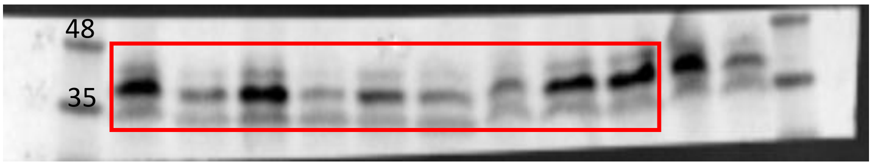

pAkt

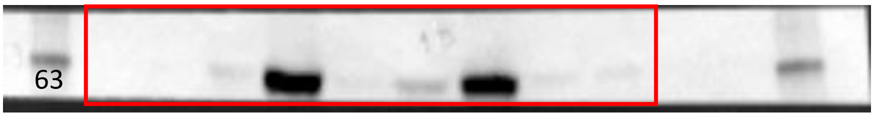

GAPDH

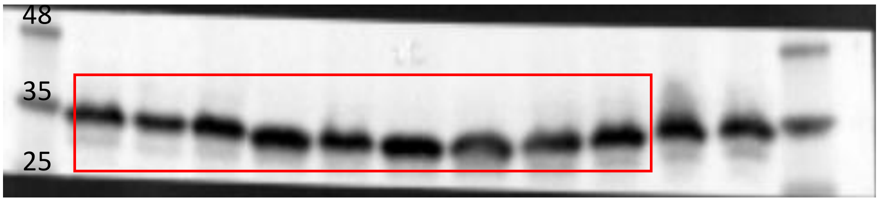

pPKM2

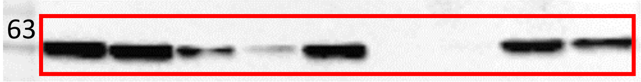

PS6K

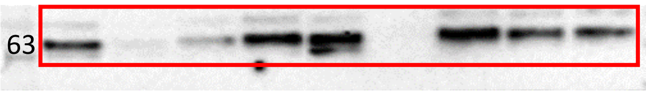

<u>Original blots for Supplementary Figure 6a:</u>

PS6K

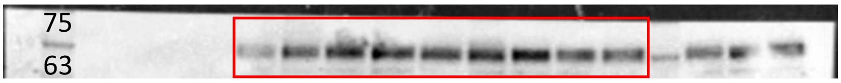

pPDGFRb

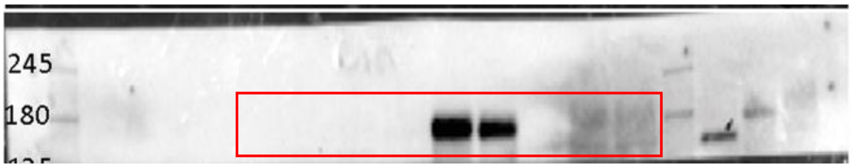

pMEK

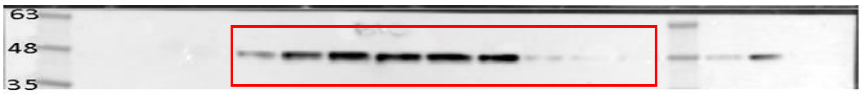

GAPDH

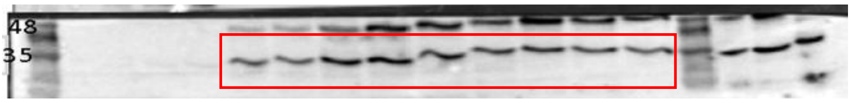

Original blots for Supplementary Figure 6b:

pS6

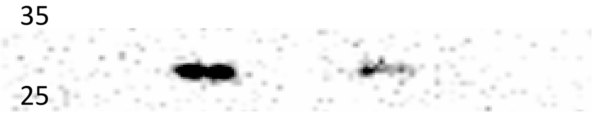

pERK

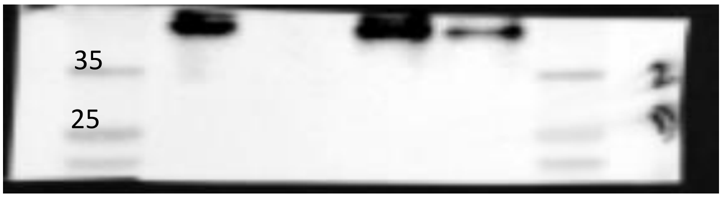

PARP

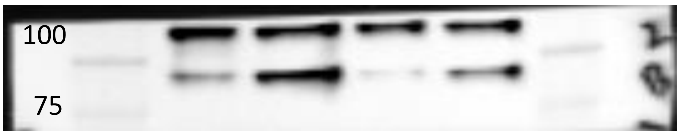

pAKT

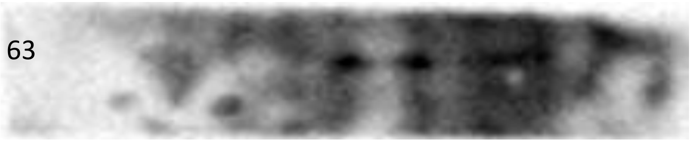

GAPDH

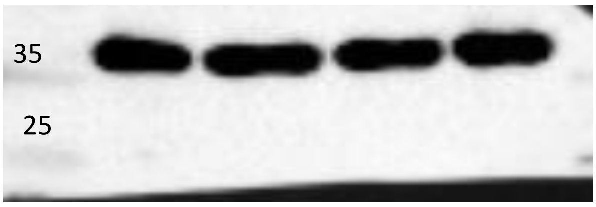

Original blots for Supplementary Figure 6c:

pPKM2

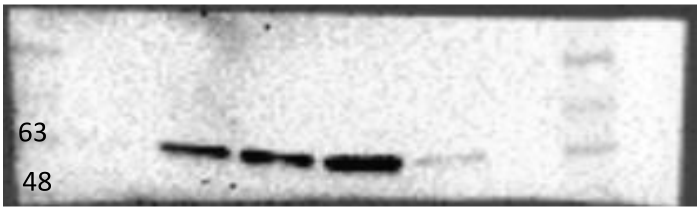

pS6

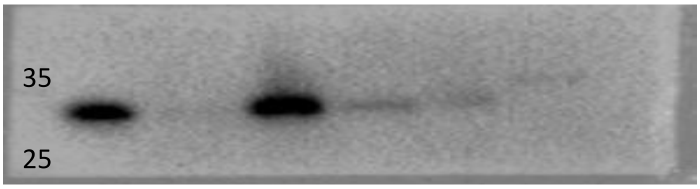

pErk

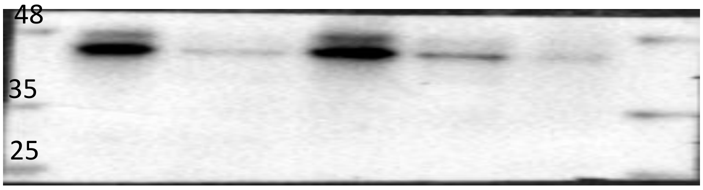

GAPDH

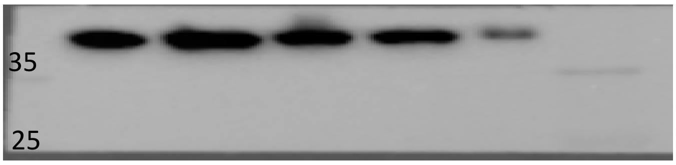

Original blots for Supplementary Figure 6d:

pS6

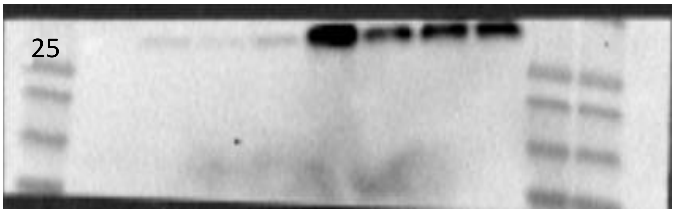

pERK2

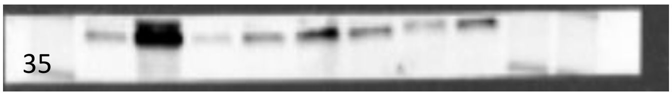

pAkt

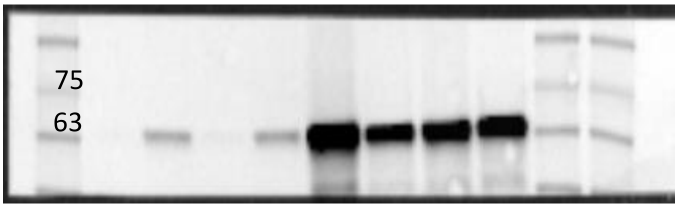

GAPDH

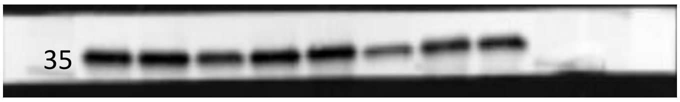

Original blots for Supplementary Figure 6e:

pS6

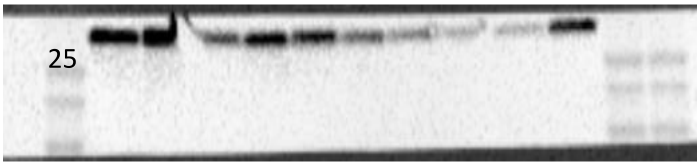

pErk2

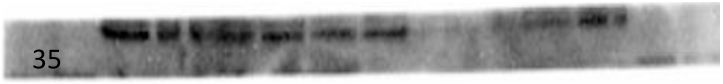

pAkt

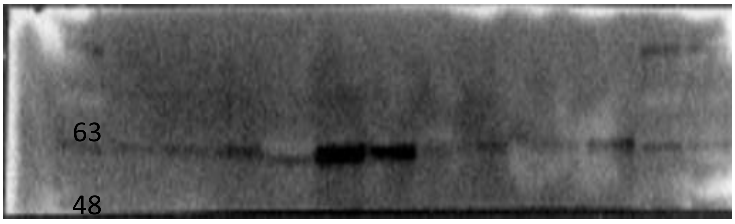

GAPDH

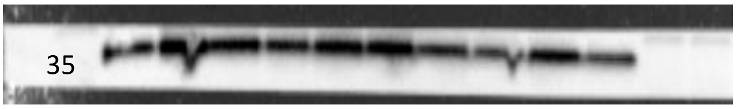

Supplementary References

1. Luo C, Shen J. Research progress in advanced melanoma. Vol. 397, Cancer Letters. Elsevier Ireland Ltd; 2017. p. 120–6.
