# Supplementary material for: Overcoming resistance to BRAFV600E inhibition in melanoma by deciphering and targeting personalized protein network alterations
Source: NPJ Precis Oncol. 2021 Jun 10;5:50. doi: 10.1038/s41698-021-00190-3 (PMC8192524; doi:10.1038/s41698-021-00190-3)
Supplement: Supplementary file 11 — Reporting Summary [file 41698_2021_190_MOESM11_ESM.pdf]

nature research

Corresponding author(s): Nataly Kravchenko-Balasha

Last updated by author(s): Feb 10, 2021



# Reporting Summary

Nature Research wishes to improve the reproducibility of the work that we publish. This form provides structure for consistency and transparency in reporting. For further information on Nature Research policies, see our Editorial Policies and the Editorial Policy Checklist.

## Statistics

For all statistical analyses, confirm that the following items are present in the figure legend, table legend, main text, or Methods section.

| n/a | Confirmed | |
|---|---|---|
| ☐ | ☒ | The exact sample size (*n*) for each experimental group/condition, given as a discrete number and unit of measurement |
| ☒ | ☐ | A statement on whether measurements were taken from distinct samples or whether the same sample was measured repeatedly |
| ☐ | ☒ | The statistical test(s) used AND whether they are one- or two-sided<br>*Only common tests should be described solely by name; describe more complex techniques in the Methods section.* |
| ☒ | ☐ | A description of all covariates tested |
| ☐ | ☒ | A description of any assumptions or corrections, such as tests of normality and adjustment for multiple comparisons |
| ☐ | ☒ | A full description of the statistical parameters including central tendency (e.g. means) or other basic estimates (e.g. regression coefficient) AND variation (e.g. standard deviation) or associated estimates of uncertainty (e.g. confidence intervals) |
| ☐ | ☒ | For null hypothesis testing, the test statistic (e.g. *F*, *t*, *r*) with confidence intervals, effect sizes, degrees of freedom and *P* value noted<br>*Give P values as exact values whenever suitable.* |
| ☒ | ☐ | For Bayesian analysis, information on the choice of priors and Markov chain Monte Carlo settings |
| ☐ | ☒ | For hierarchical and complex designs, identification of the appropriate level for tests and full reporting of outcomes |
| ☐ | ☒ | Estimates of effect sizes (e.g. Cohen's *d*, Pearson's *r*), indicating how they were calculated |

*Our web collection on statistics for biologists contains articles on many of the points above.*

## Software and code

Policy information about availability of computer code

| | |
|---|---|
| Data collection | No Software was used. |
| Data analysis | All calculations were performed based on the the equations published and available in Vasudevan et al., PNAS 2018 and Flashner-Abramson et al., Theranostics, 2019, and Kravchenko-Balasha et al., 2011, BMC Systems Biology. |

For manuscripts utilizing custom algorithms or software that are central to the research but not yet described in published literature, software must be made available to editors and reviewers. We strongly encourage code deposition in a community repository (e.g. GitHub). See the Nature Research guidelines for submitting code & software for further information.

## Data

Policy information about availability of data

All manuscripts must include a data availability statement. This statement should provide the following information, where applicable:

- Accession codes, unique identifiers, or web links for publicly available datasets
- A list of figures that have associated raw data
- A description of any restrictions on data availability

The patient dataset were selected from a large TCPA dataset containing 7694 cancer tissues from various anatomical origins (PANCAN32, level 4) . The dataset for the cancer cell lines was downloaded from the TCPA portal. The data was already published by Li et al.

# Field-specific reporting

Please select the one below that is the best fit for your research. If you are not sure, read the appropriate sections before making your selection.

☒ Life sciences ☐ Behavioural & social sciences ☐ Ecological, evolutionary & environmental sciences

For a reference copy of the document with all sections, see nature.com/documents/nr-reporting-summary-flat.pdf

# Life sciences study design

All studies must disclose on these points even when the disclosure is negative.

| | |
|---|---|
| Sample size | In vitro - In each experiment, every condition or sample has triplicates. Invivo - 7-8 mice were used for each condition to make the experiment statistically significant. Student t.test was used to determine the p values of different conditions. |
| Data exclusions | No data was excluded. |
| Replication | Each experiment was repeated at least 2 times. The data was reproducible every time. |
| Randomization | In vivo - 40 mice for every cell lines were all injected with tumors and were randomly distributed to each group based on the tumor size. |
| Blinding | Blinding was not possible since each group of mice were treated with a different therapy. |

# Reporting for specific materials, systems and methods

We require information from authors about some types of materials, experimental systems and methods used in many studies. Here, indicate whether each material, system or method listed is relevant to your study. If you are not sure if a list item applies to your research, read the appropriate section before selecting a response.

## Materials & experimental systems

| n/a | Involved in the study | |
|---|---|---|
| ☐ | ☒ | Antibodies |
| ☐ | ☒ | Eukaryotic cell lines |
| ☒ | ☐ | Palaeontology and archaeology |
| ☐ | ☒ | Animals and other organisms |
| ☒ | ☐ | Human research participants |
| ☒ | ☐ | Clinical data |
| ☐ | ☒ | Dual use research of concern |

## Methods

| n/a | Involved in the study | |
|---|---|---|
| ☒ | ☐ | ChIP-seq |
| ☒ | ☐ | Flow cytometry |
| ☒ | ☐ | MRI-based neuroimaging |

## Antibodies

| | |
|---|---|
| Antibodies used | Anti-p-PKM2 (Tyr105) (cat. no. 3827S; 1:1000), anti-p-S6 (Ser235/236) (cat. no. 4858S; 1:1000), anti-p-PDGFRβ (Y751) (cat. no. 4549S; 1:1000), anti-p- AKT (Ser473) (cat. no. 4060S; 1:1000), anti-p-p70S6K (Tyr389) (cat. no. 9205L; 1:1000), anti-p-Mek (Ser217/221) (cat. no. 9154S ; 1:1000) and anti-total –PARP (cat. no. 9542S; 1:1000) antibodies were purchased from Cell Signaling Technology, Inc. Anti-p-ERK2 (E4) (cat. no. SC7383; 1:200), anti-total -P53 (cat. no. SC126; 1:200) and anti-total -GAPDH (cat. no. SC47724; 1:200) antibodies were purchased from Santa Cruz Biotechnology. |

Validation

1. p-PKM2 - cat no: 3827S :
MW (kDa) 60
SOURCE Rabbit
Phosphorylation of PKM2 on Tyr105 was identified at Cell Signaling Technology (CST) using PhosphoScan®, CST's LC-MS/MS platform for phosphorylation site discovery.
Christofk, H.R. et al. (2008) Nature 452, 230-3.
Mazurek, S. et al. (2005) Semin Cancer Biol 15, 300-8.
CITATION: Role of pyruvate kinase M2-mediated metabolic reprogramming during podocyte differentiation.Qi Yuan, et. al.Cell Death Dis, 2020.

2. p-S6 - cat. no: 4858S:
MW (kDa) 32
Source/Isotype Rabbit IgG
Monoclonal antibody is produced by immunizing animals with a synthetic phosphopeptide corresponding to residues surrounding Ser235 and Ser236 of human ribosomal protein S6.
Dufner, A. and Thomas, G. (1999) Exp Cell Res 253, 100-9.
Peterson, R.T. and Schreiber, S.L. (1998) Curr Biol 8, R248-50.

CITATION: Comp34 displays potent preclinical antitumor efficacy in triple-negative breast cancer via inhibition of NUDT3-AS4, a no...Qiongyu Hao, et. al.Cell Death Dis, 2020.

3. p-PDGFRb - cat. no : 4549S:
MW (kDa) 190
Source/Isotype Rabbit IgG
Monoclonal antibody is produced by immunizing animals with a synthetic phosphopeptide corresponding to residues surrounding Tyr751 of human PDGF receptor β.
Deuel, T.F. et al. (1988) Biofactors 1, 213-217.
Bergsten, E. et al. (2001) Nat. Cell Biol. 3, 512-516.
CITATIONS: Interplay between c-Src and the APC/C co-activator Cdh1 regulates mammary tumorigenesis.Tao Han, et. al. Nat Commun, 2019.

4.p-AKT - cat no: 4060S:
MW (kDa) 60
Source/Isotype Rabbit IgG
Monoclonal antibody is produced by immunizing animals with a synthetic phosphopeptide corresponding to residues around Ser473 of human Akt.
Franke, T.F. et al. (1997) Cell 88, 435-7.
Burgering, B.M. and Coffer, P.J. (1995) Nature 376, 599-602.
Franke, T.F. et al. (1995) Cell 81, 727-36.
CITATIONS:OxLDL/β2GPI/anti-β2GPI Ab complex induces inflammatory activation via the TLR4/NF-κB pathway in HUVECs. Guiting Zhang, et. al.Mol Med Rep, 2021

5. p-p70S6K - cat no : 9205L :
MW (kDa) 70, 85
SOURCE Rabbit
Polyclonal antibodies are produced by immunizing animals with a synthetic phosphopeptide corresponding to residues around Thr389 of human p70 S6 kinase. Antibodies are purified by protein A and peptide affinity chromatography.
Pullen, N. and Thomas, G. (1997) FEBS Lett 410, 78-82.
Dufner, A. and Thomas, G. (1999) Exp Cell Res 253, 100-9.
Weng, Q.P. et al. (1998) J Biol Chem 273, 16621-9.
CITATIONS: Selective inhibition of PI3K110α as a novel therapeutic strategy for cetuximab-resistant oral squamous cell carcinoma., Hiroki Tsuchihashi, et. al. Oncol Rep, 2020.

6. p-MEK - cat no : 9154S:
MW (kDa) 45
Source/Isotype Rabbit
Monoclonal antibody is produced by immunizing animals with a synthetic phosphopeptide corresponding to residues around Ser217/221 of human MEK1/2.
Crews, C.M. et al. (1992) Science 258, 478-480.
Alessi, D.R. et al. (1994) EMBO J. 13, 1610-19.
CITATIONS: LEF1/Id3/HRAS axis promotes the tumorigenesis and progression of esophageal squamous cell carcinoma.Xinyu Wang, et. al. Int J Biol Sci, 2020.

7. PARPV- cat no : 9542S:
MW (kDa) 89, 116
SOURCE Rabbit
Polyclonal antibodies are produced by immunizing animals with a synthetic peptide corresponding to the caspase cleavage site in PARP. Antibodies are purified by protein A and peptide affinity chromatography.
Satoh, M.S. and Lindahl, T. (1992) Nature 356, 356-358.
Lazebnik, Y. A. et al. (1994) Nature 371, 346-347.
Cohen, G.M. (1997) Biochem J 326 ( Pt 1), 1-16.
CITATIONS:
CHD7 and 53BP1 regulate distinct pathways for the re-ligation of DNA double-strand breaks. Magdalena B Rother, et. al. Nat Commun,2020.

8. p-ERK2 - cat no: SC7383 :
MW (kDa) 42, 44
SOURCE Mousep-ERK (E-4) is a mouse monoclonal antibody epitope corresponding to a  sequence containing Tyr 204 phosphorylated ERK of human origin. Boulton, T., et al. 1991. ERKs: a family of protein-serine/threonine kinases that are activated and tyrosine phosphorylated in response to Insulin and NGF. Cell 65: 663-675. Boulton, T.G., et al. 1991. Purification and properties of ERK 1, an Insulinstimulated MAP2 protein kinase. Biochemistry 30: 278-286.
CITATIONS: 1. Kabarowski, J.H., et al. 2001. Lysophosphatidylcholine as a ligand for the immunoregulatory receptor G2A. Science 293: 702-705.
2. Mercurio, L., et al. 2018. IL-38 has an anti-inflammatory action in psoriasis and its expression correlates with disease severity and therapeutic response to anti-IL-17A treatment. Cell Death Dis. 9: 1104.

9. p53 - cat no : SC126:
MW (kDa) 53
SOURCE Mouse
p53 (DO-1) is a mouse monoclonal antibody epitope mapping between amino acid residues 11-25 at the N-terminus of p53 of human origin.
Banks, L., et al. 1986. Isolation of human-p53-specific monoclonal antibodies and their use in the studies of human p53 expression. Eur. J.Biochem. 159: 529-534.
Hupp, T.R., et al. 1992. Regulation of the specific DNA binding function of p53. Cell 71: 875-886.

CITATIONS: . Schmoldt, A., et al. 1975. Digitoxin metabolism by rat liver microsomes. Biochem. Pharmacol. 24: 1639-1641.
Lee, J.W., et al. 2019. RUNX3 regulates cell cycle-dependent chromatin dynamics by functioning as a pioneer factor of the restriction-point. Nat. Commun. 10: 1897.

10. GAPDH - cat no : SC47724:
MW (kDa) 37
SOURCE Mouse
GAPDH (0411) is a mouse monoclonal antibody raised against recombinant GAPDH of human origin.
CITATIONS: . Daling, J.R., et al. 1987. Sexual practices, sexually transmitted diseases, and the incidence of anal cancer. N. Engl. J. Med. 317: 973-977.
Smith, R.L., et al. 2014. Synthesis of a novel legumain-cleavable colchicine prodrug with cell-specific toxicity. Bioorg. Med. Chem. 22: 3309-3315.
Zhang, Y., et al. 2015. Down-regulated long non-coding RNA MEG3 and its effect on promoting apoptosis and suppressing migration of trophoblast cells. J. Cell. Biochem. 116: 542-550.

## Eukaryotic cell lines

Policy information about cell lines

| | |
|---|---|
| Cell line source(s) | ATCC |
| Authentication | The cell lines were authenticated at the Biomedical Core Facility of the Technion, Haifa, Israel. |
| Mycoplasma contamination | No Mycoplasma contamination was found |
| Commonly misidentified lines (See ICLAC register) | All the cell lines used were BRAF mutated cell lines. |

## Animals and other organisms

Policy information about studies involving animals; ARRIVE guidelines recommended for reporting animal research

| | |
|---|---|
| Laboratory animals | NSG mice, males around 6-8 weeks old were used. |
| Wild animals | Study did not use wild animals. |
| Field-collected samples | Study did not use field collected samples |
| Ethics oversight | The Hebrew University is an AAALAC International accredited institute. All experiments were conducted with approval from the Hebrew University Animal Care and Use Committee. Ethical accreditation number: Md-17-15174-4. |

Note that full information on the approval of the study protocol must also be provided in the manuscript.

## Dual use research of concern

Policy information about dual use research of concern

### Hazards

Could the accidental, deliberate or reckless misuse of agents or technologies generated in the work, or the application of information presented in the manuscript, pose a threat to:

| No | Yes | |
|---|---|---|
| ☒ | ☐ | Public health |
| ☒ | ☐ | National security |
| ☒ | ☐ | Crops and/or livestock |
| ☒ | ☐ | Ecosystems |
| ☒ | ☐ | Any other significant area |

## Experiments of concern

Does the work involve any of these experiments of concern:

| No | Yes | |
|---|---|---|
| ☒ | ☐ | Demonstrate how to render a vaccine ineffective |
| ☒ | ☐ | Confer resistance to therapeutically useful antibiotics or antiviral agents |
| ☒ | ☐ | Enhance the virulence of a pathogen or render a nonpathogen virulent |
| ☒ | ☐ | Increase transmissibility of a pathogen |
| ☒ | ☐ | Alter the host range of a pathogen |
| ☒ | ☐ | Enable evasion of diagnostic/detection modalities |
| ☒ | ☐ | Enable the weaponization of a biological agent or toxin |
| ☒ | ☐ | Any other potentially harmful combination of experiments and agents |
